# Assisting an Australian Aboriginal and Torres Strait Islander person with gambling problems: a Delphi study

**DOI:** 10.1186/s40359-017-0196-x

**Published:** 2017-08-02

**Authors:** Kathy S Bond, Katrina M. Dart, Anthony F. Jorm, Claire M. Kelly, Betty A. Kitchener, Nicola J. Reavley

**Affiliations:** 1 Mental Health First Aid Australia, Level 6/369 Royal Parade, Parkville, VIC 3052 Australia; 2 Centre for Mental Health, Melbourne School of Population and Global Health, The University of Melbourne, Level 4/207 Bouverie Street, Parkville, VIC 3010 Australia; 3 School of Psychology, Deakin University, 1 Gheringhap Street, Geelong, VIC 3220 Australia

**Keywords:** Aboriginal, Torres Strait Islander, Gambling problems, Mental health first aid, How to assist

## Abstract

**Background:**

Gambling problems appear to be more prevalent in the Australian Aboriginal and Torres Strait Islander population than in the non-Indigenous population. Although gambling harms can be significant, treatment-seeking rates are low. The Delphi expert consensus method was used to develop a set of guidelines on how a family or community member can assist an Aboriginal or Torres Strait Islander person with gambling problems.

**Methods:**

Building on a previous systematic review of websites, books and journal articles a questionnaire was developed that contained items about the knowledge, skills and actions needed for supporting an Aboriginal or Torres Strait Islander person with gambling problems. These items were rated over three rounds by an expert panel comprising professionals who provide treatment to or conduct research with Aboriginal and Torres Strait Islander people with gambling problems.

**Results:**

A total of 22 experts rated 407 helping statements according to whether they thought the statements should be included in these guidelines. There were 225 helping statements that were endorsed by at least 90% of participants. These endorsed statements were used to develop the guidelines.

**Conclusion:**

Experts were able to reach substantial consensus on how someone can recognise the signs of gambling problems and support an Aboriginal or Torres Strait Islander person to change.

**Electronic supplementary material:**

The online version of this article (doi:10.1186/s40359-017-0196-x) contains supplementary material, which is available to authorized users.

## Background

Gambling problems appear to be more prevalent in the Australian Aboriginal and Torres Strait Islander population than in the non-Indigenous population [1–3], with an Australia-wide study finding that Indigenous Australians reported gambling problems in themselves or someone they know at three to four times the rate of non-Indigenous Australians [4].

Rates of treatment seeking for gambling problems in the general Australian population are low (19.2%) [5], with rates increasing with the severity of the problems [6]. Although Australian research is sparse, a study in the state of New South Wales indicates that help seeking for gambling problems in Aboriginal and Torres Strait Islander people is even lower (8.8%) [7]. The identified barriers to treatment seeking in Aboriginal and Torres Strait Islander people are recognising that their gambling is a problem, shame and stigma associated with gambling problems and a lack of culturally appropriate gambling help services [7, 8]. The strongest motivators for help-seeking in the general population are related to the harms associated with gambling (e.g. relationship problems, problems with housing and legal problems) and “pressure from family or friends”. Furthermore, professional help-seeking is often preceded and followed by informal help-seeking, e.g. seeking help from family or friends [8]. Research with Indigenous populations in Australia suggests that encouragement from family and friends can facilitate treatment seeking for gambling problems [9]. However, for family and friends to be able to encourage someone to seek help for gambling problems, they must be able to recognise that there is a problem.

Gambling problems are defined as gambling activities where the person struggles to limit the amount of money or time spent on gambling [10]. However, these characteristics are not necessarily overt, potentially meaning that gambling problems are hidden from family members, friends and co-workers. When the problems do become evident, family and friends may not feel as if they know how to talk about gambling problems with the person. Research in Indigenous populations in Australia and internationally suggests that family, friends and the community can be a great source of help for gambling problems, if they have appropriate knowledge about how to recognise and support someone with gambling problems [7, 11].

Provision of guidelines on assisting a person with gambling problems and attending training courses are two potential interventions that can teach family and community members to recognise the signs of gambling problems in a person and support them to change. Guidelines developed using the Delphi expert consensus method, are available on how members of the public can recognise and assist an Aboriginal or Torres Strait Islander person who has a mental health problem or is in a mental health crisis [12–14]. These guidelines have been used as the basis for the 14 h Aboriginal Mental Health First Aid Australia (AMHFA) training course [15]. The AMHFA course has been evaluated using focus group methodology and was found to be “culturally appropriate, empowering for Indigenous people, and provided information that was seen as highly relevant and important in assisting Aboriginal people with a mental illness” [16].

A recent Delphi study was conducted to develop guidelines for how to help a friend, family member or co-worker who had gambling problems [17, 18]. This study identified the signs that may indicate a person has a gambling problem and outlines what a person needs to know and do to help them. Because the purpose of this study was to develop guidelines for a broad range of English-speaking, Western countries, it is not known if they are culturally appropriate for or applicable to Australian Aboriginal and Torres Strait Islander people. Therefore, this study aimed to develop mental health first aid guidelines on how to assist an Aboriginal or Torres Strait Islander person with gambling problems. Specifically, we aimed to: (1) determine, using the Delphi method, how members of the community can best assist an Aboriginal or Torres Strait Islander person who has gambling problems; (2) develop a list of evidence-informed, observable signs that a member of the public can use to help identify an Aboriginal or Torres Strait Islander person who may have gambling problems; and (3) produce a guidelines document that is available to the public and that will inform future Mental Health First Aid training.

## Methods

The Delphi method [19] is a way of determining the consensus of a group of experts on a particular topic. It is particularly helpful in developing guidelines where the use of other research methods are not appropriate, e.g. randomised controlled trials. Development of the current guidelines involved four steps: (1) formation of the expert panel, (2) literature search and survey development, (3) data collection and analysis, and (4) guidelines development.

### Step 1: Panel formation

In line with other similar Aboriginal and Torres Strait Islander mental health first aid Delphi studies (e.g. [12]), this study utilised one expert panel consisting of professionals with experience researching or treating gambling problems in Aboriginal and Torres Strait Islander people. The decision to use only one expert panel was made because the field of Aboriginal and Torres Strait Islander gambling is small and it was thought that it would have been difficult to recruit enough Aboriginal and Torres Strait Islander people to a ‘lived experience’ and ‘affected other’ panel to produce meaningful results. Therefore, the selection criteria were:

- 18 years or over, AND
- A gambling help professional or researcher who is informed about Aboriginal and Torres Strait Islander gambling, AND
- Have a minimum of 2 years’ experience in the field of Aboriginal and Torres Strait Islander gambling problems.

The aim was to recruit a minimum of 30 people to the panel, which is within the typical Delphi panel size of 15–60 experts [20], allowing for reliable consensus to be reached.

Twenty-two participants with experience in working with Aboriginal and Torres Strait Islander people with gambling problems completed all three rounds. The participants had an average age of 46.4 (SD 10.48) and 12 were female and ten male. They were from the following Australian states and territories: ACT (*n* = 1), NSW (*n* = 2), SA (*n* = 1), VIC (*n* = 7), NT (*n* = 2), QLD (*n* = 6), TAS (*n* = 1) and WA (*n* = 2). They worked in an Aboriginal gambling service (*n* = 7), a general gambling service (*n* = 5), a local health service (*n* = 4), a community service (*n* = 1), or other mental health setting (*n* = 3), or as a researcher (*n* = 4) (note: some may have worked in more than one service). Seven of the participants were Aboriginal and none were Torres Strait Islander. Three participants had experienced gambling problems themselves, while 10 had supported a family member and 11 a fellow community member who experienced gambling problems. The retention rate for participants completing all three rounds was 84.6% (26 participants completed Round 1).

### Step 2: Literature search and survey development

The Round 1 survey included two types of questions – items from a previous international study to develop guidelines for assisting people with gambling problems in developed English-speaking Western countries [17] and items derived from a targeted literature search described below. The methodology of the study to develop the gambling guidelines for English-speaking Western countries is described in detail elsewhere [17]. Three hundred and forty-seven items from the previous survey that received a consensus rating of at least 50% were used in the Round 1 survey of this current study.

In order to further inform the content of the initial survey, a systematic search of the ‘grey’ and academic literature was conducted in July 2015 to gather statements about how to help an Aboriginal or Torres Strait Islander person with gambling problems. The website search was conducted using Google Australia, the book search was conducted using Google Books and the journal search was conducted using Google Scholar and PubMed. See Table 1 for the search terms.

**Table 1:** Search terms

Search type	Search source	Search terms
Websites and Books	Google Australia and Google Books	• (Australian Aboriginal OR Australian Indigenous OR Torres Strait Islander) AND (gambling) • (Australian Aboriginal OR Australian Indigenous OR Torres Strait Islander AND problem gambling • (Australian Aboriginal OR Australian Indigenous OR Torres Strait Islander) AND (pathological gambling) • (Australian Aboriginal OR Australian Indigenous OR Torres Strait Islander) AND (gambling addiction) • (Australian Aboriginal OR Australian Indigenous OR Torres Strait Islander) AND (compulsive gambling) • (Helping someone who gambles) AND (Australian Aboriginal OR Australian Indigenous OR Torres Strait Islander) • (Helping someone stop gambling) AND (Australian Aboriginal OR Australian Indigenous OR Torres Strait Islander)
Journal articles	Google Scholar	• (Helping someone who gambles) AND (Australian Aboriginal OR Australian Indigenous OR Torres Strait Islander) • (Helping someone stop gambling) AND (Australian Aboriginal OR Australian Indigenous OR Torres Strait Islander)
	PubMed	• (Aboriginal OR Indigenous OR Torres Strait Islander) AND (Australian) AND (gambling) • (Aboriginal OR Indigenous OR Torres Strait Islander) AND (Australian) AND (problem gambling) • (Aboriginal OR Indigenous OR Torres Strait Islander) AND (Australian) AND (pathological gambling) • (Aboriginal OR Indigenous OR Torres Strait Islander) AND (Australian) AND (compulsive gambling)

**Fig. 1:**
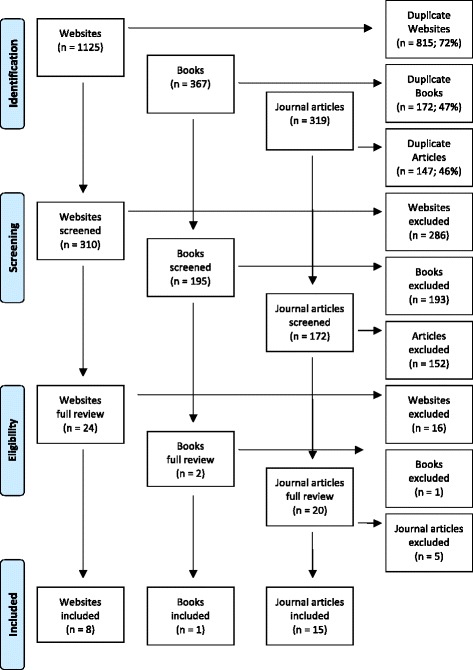
Literature search

In line with other similar Delphi studies (e.g. [17, 21, 22]), the first 50 websites, 50 books and 50 journal articles were retrieved. The decision to examine the first 50 for each search term was based on a previous Delphi study that found that the quality of the resources declined rapidly after the first 50 [23]. After duplicates were removed, the remaining sources were reviewed for relevant information, as were any links appearing on the websites. Websites, articles and books were excluded if they did not contain information about how a member of the public can recognise and help an Aboriginal or Torres Strait Islander person who has gambling problems. A total of 24 websites, articles and books were included and used to develop the Round 1 survey. Figure 1 summarises the results of the literature search.

A working group, consisting of staff from Mental Health First Aid Australia, the University of Melbourne and an Aboriginal and Torres Strait Islander mental health first aid expert (who is Aboriginal) translated the results from the literature search into helping statements that were clear, actionable, and contained only one idea. These statements, plus the items from the recent Delphi study to develop guidelines for helping a person with gambling problems from a developed English-speaking Western country [17], were used to form the first of three questionnaires that were administered to the expert panel via SurveyMonkey.

In this study, a distinction was made between the subclinical symptoms of problem gambling and *gambling disorder* (a diagnosis). We use the term *gambling problems*, defined as gambling activities where the person struggles to limit the amount of time or money spent on gambling, leading to adverse consequences for the person, their family, or the community. This includes someone whose gambling problems are at a clinically diagnosable level [10]. This definition was used because it is not feasible or preferred that members of the public (e.g. family or friends) diagnose pathological or disordered gambling, and because the study sought to identify the signs of a range of gambling problems (from at risk gambling through to problem gambling). Also, if family, friends and co-workers can recognise, identify and address gambling problems earlier, severe gambling harms may be prevented.

### Step 3: Data collection and analysis

Data were collected in a survey administered over three rounds between January and April 2016. In the survey, participants were asked to rate each of the helping statements, using a 5-point scale (‘essential’, ‘important’, ‘don’t know/depends’, ‘unimportant’ or ‘should not be included’), according to whether or not they thought the statement should be included in the guidelines. In Round 1, participants also could provide qualitative data in the form of suggestions for new helping statements. See Additional file 1 for copies of the three rounds of the survey.

The statements were analysed and categorised as follows:

1. Endorsed. The item received an ‘essential’ or ‘important’ rating from 90 to 100% of participants.
2. Re-rate. The item received an ‘essential’ or ‘important’ rating from 80 to 89% of participants.
3. Rejected. The item did not fall into either the endorsed or re-rate categories.

These cut-off criteria were chosen by the working group because there was only one panel and lower cut-off percentages would have yielded too many statements, making the guidelines impractical to use.

The following criteria were used to determine whether the participants’ comments would be translated into new helping statements: (1) the idea was actionable and understandable, (2) it was not a repeat of an item in the first survey, and (3) it was within the scope of the project. This new content was translated into helping statements for the Round 2 survey. The Round 2 survey also included Round 1 items that needed to be re-rated. Participants were given a summary of Round 1 that included a list of the items that were endorsed and rejected, as well as the items that needed to be re-rated in Round 2. The summary included the panel percentages of each rating, as well as the specific panel member’s scores for each re-rated item. This allowed the participants to compare their ratings with the expert panel’s consensus rating and decide if they wanted to maintain or change their answer when re-rating an item.

The procedures for Rounds 2 and 3 were the same as Round 1 with several exceptions. Round 2 consisted of new items from the Round 1 comments. There was no opportunity for comments in Round 2 or Round 3, and if a re-rated item did not receive an ‘essential’ or ‘important’ rating by 90% or more of the panel, it was rejected. Round 3 only contained items introduced in Round 2 that needed to be re-rated, according to the above criteria.

### Step 4: Guidelines development

The endorsed items were written into continuous prose to form the guidelines. The first author drafted the guidelines and the working group edited the draft to produce the final guidelines document. This document was presented to the expert participants for comment and final endorsement.

## Results

**Fig. 2:**
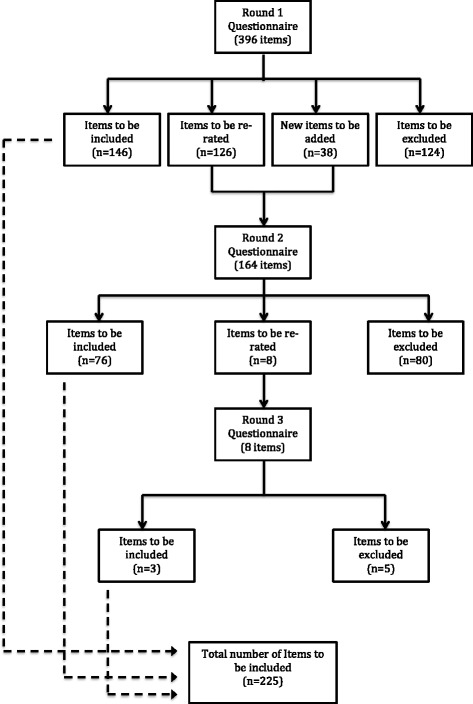
Rated items

A total of 407 items were rated over three rounds to yield a total of 225 endorsed items. Figure 2 presents the information about the total number of items rated, endorsed and rejected, and Additional file 2 presents the endorsement or rejection percentages of each item. The endorsed items formed the basis of the guidelines document.

The endorsed items outlined what Aboriginal, Torres Strait Islander and non-Indigenous members of the community need to know and do to support an Aboriginal or Torres Strait Islander person with gambling problems. This includes knowing about gambling and gambling problems, the association between mental health problems and gambling problems, and the signs that may indicate a person has gambling problems. The guidelines also present specific actions for approaching and talking with the person in a non-judgmental way. Effective ways of encouraging change and help-seeking are presented, as well as ways to support the person even if they do not wish to change their gambling. Strategies for managing crisis situations (e.g. suicide) are also covered. Importantly, the information and actions suggested in the guidelines are presented within a context of cultural competence and safety.

### Guidelines development

The first author grouped items thematically under specific headings, re-writing them into continuous prose for ease of reading. As much as possible, original wording of the items was retained. Some examples and explanatory notes to clarify the advice were given, for example, the risk factors for gambling problems were included in the guidelines. The draft guidelines were then presented to participants for final comment, feedback and endorsement. All participants endorsed the guidelines without suggesting changes.

The final guidelines (available at: mhfa.com.au/resources/mental-health-first-aid-guidelines) provide information on how to assist an Aboriginal or Torres Strait Islander person with gambling problems [24]. The main themes and subthemes follow:

- What are gambling problems?
- Gambling problems and Aboriginal culture.
- Motivations for gambling.
- How can I tell if someone has gambling problems?
- Gambling behaviours.
- Signs evident while gambling.
- Mental and physical health signs.
- Financial signs.
- Social signs.
- Signs evident at home (which includes signs that may be evident in family members).
- Signs evident in the workplace.
- Approaching someone about their gambling.
- How to talk to the person.
- Dealing with negative reactions.
- Encouraging professional help.
- Encouraging the person to change.
- If the person does not want to change**.**
- Supporting the person to change.
- What to do if you are concerned for the safety of the person or others.

## Discussion

This research aimed to develop a set of guidelines on how to assist an Aboriginal or Torres Strait Islander person with gambling problems. Overall, 225 items were endorsed by the expert panel as being important or essential to the guidelines. The endorsed items were written into a guidelines document that is available to the public via the Mental Health First Aid Australia website (mhfa.com.au/resources/mental-health-first-aid-guidelines).

A strength of the guidelines is that they address a wide variety of topics or situations that a person may encounter when supporting an Aboriginal or Torres Strait Islander person with gambling problems. These include recognising the warning signs of gambling problems, talking to a person if you are concerned that they have gambling problems, encouraging the person to change (including specific strategies to reduce gambling harms) and what to do if the person is resistant to changing their gambling.

### Comparison between these guidelines and the guidelines for people from English-speaking Western countries

Recently, guidelines for assisting a person with gambling problems from English-speaking Western countries have been published [17, 18]. There are some notable differences between these two sets of guidelines (see Additional file 3 for a comparison of the two sets of items). The Aboriginal and Torres Strait Islander guidelines include a section on the impact of culture on gambling problems. This section includes more general information about Aboriginal and Torres Strait Islander understanding of health and well-being, as well as specific social expectations that may impact on gambling problems. For instance, the expert panellists determined that the first aider should know about the Aboriginal and Torres Strait Islander expectation that one provides for their family and kin. It is important that the first aider know this because this cultural norm may mean that the effects of gambling are felt more widely throughout the community or that gambling harms are lessened, reducing the motivation to change [25].

Another difference is in the way the first aider should approach the person. In the guidelines for English-speaking Western countries, the first aider should “First state some positive things about the person and your relationship with them” and then “… talk about what behaviours you have noticed…”. This is a more direct approach than what is recommended if you are concerned about an Aboriginal or Torres Strait Islander person, where a ‘gentle’ and ‘gradual’ approach is recommended, including having a “yarn (discussion) about other topics to try to find some common ground for discussion” before introducing concerns about gambling.

The most notable difference between these guidelines and the guidelines for English-speaking Western countries is in the number of items related to money that were endorsed. The survey had items relating to financial warning signs (e.g. complaints about mounting debt) and financial strategies for reducing the impact of gambling (e.g. leaving cash at home when going gambling). Of the financial signs that were rated, 67% were endorsed in the previous survey, while only 41% were endorsed in the Aboriginal and Torres Strait Islander survey. It may be that because Aboriginal and Torres Strait Islander people experience economic disadvantage at higher rates than non-Indigenous people [26], some of the financial signs may be indicators of this general economic disadvantage rather than of gambling problems specifically. Furthermore, 80% per cent of the financial strategies to reduce the impact of gambling were endorsed in the survey for English-speaking Western countries as compared to only 25% for the Aboriginal and Torres Strait Islander survey. Examples of the financial strategies include leaving cash at home when going gambling and allowing someone else to temporarily manage accounts or money. A number of social factors may be responsible for these items not being endorsed in the Aboriginal and Torres Strait Islander study. Household overcrowding, particularly in rural and remote households, may mean that if money is left at home it may be used by others in the household [4, 27]. Also money is often seen as a shared resource rather than belonging to any one individual in the family or household [28, 29], making some of the items about giving someone else control over your money irrelevant. Finally, poor financial literacy for some Aboriginal and Torres Strait Islander people and limited access to financial services for rural and remote people [28] may mean some of these items are not applicable to some Aboriginal and Torres Strait Islander people, e.g. “Increases their usage of or acquires additional credit cards” as a warning sign for gambling problems.

This research has a few limitations. First, there is limited research that indicates what is most helpful for Aboriginal and Torres Strait Islander people with gambling problems; therefore, limiting the initial literature search. However, because participants could suggest missing helping actions, this limitation should be minimised. Another limitation is the possibility that some participants rated statements that were beyond their expertise, leading to an omission of useful items. Furthermore, participants were not able to discuss their comments with others. If panel members held biases or incorrect assumptions that were unchallenged because there was no opportunity for discussion, it is possible that key actions were omitted from the guidelines. The panel size for this research is another potential limitation – despite extensive recruitment only 22 people completed all three surveys. Akins, Tolson and Cole [30] found that “Panels of similarly trained experts (who possess a general understanding in the field of interest) provide effective and reliable utilization of a small sample from a limited number of experts (in this case 23) in a field of study to develop reliable criteria that inform judgment and support effective decision-making.” The use of only one panel of experts (professionals) could be seen as a limitation. However, a majority of the participants had experienced gambling problems in themselves, a family member or community member giving them ‘lived experience’ expertise as well. Finally, although it was the intent to recruit Torres Strait Islander people to the expert panel, we were unable to do this and this may limit the applicability of these guidelines to this population.

Future research to develop guidelines for helping Aboriginal and Torres Strait Islander family and community members affected by another person’s gambling would be beneficial. Also, research could evaluate the perceived usefulness of downloading and reading these guidelines, as has previously been done for other sets of mental health first aid guidelines by Hart and colleagues [31]. Furthermore, research could be conducted to validate the identified signs of gambling problems or the effectiveness of the actions suggested in the guidelines. Finally, any courses that are developed using these guidelines should be evaluated.

## Conclusion

Gambling problems cause significant harms in Aboriginal and Torres Strait Islander communities. The guidelines developed in this current study will provide needed guidance on how to assist an Aboriginal or Torres Strait Islander person with gambling problems. Professionals who treat Aboriginal and Torres Strait Islander people with gambling problems were able to reach consensus about a number of strategies for assisting an Aboriginal or Torres Strait Islander person with gambling problems. It is anticipated that these guidelines will inform future training and will be used by individuals to support people with gambling problems.

## Additional files

Additional file 1: Surveys: Copies of the three rounds of the survey (PDF 2627 kb)

Additional file 2: Rated Items: Endorsement or rejection percentages of each item (XLSX 54 kb)

Additional file 3: Comparisons: Comparison of the endorsed Aboriginal items to the English-speaking Western countries items. (XLSX 50 kb)

## Abbreviations

AMHFA: Aboriginal and Torres Strait Islander Mental Health First Aid
