# Supplementary material for: Assisting an Australian Aboriginal and Torres Strait Islander person with gambling problems: a Delphi study
Source: BMC Psychol. 2017 Aug 2;5:27. doi: 10.1186/s40359-017-0196-x (PMC5541654; doi:10.1186/s40359-017-0196-x)
Supplement: Supplementary file 1 — Surveys: Copies of the three rounds of the survey (PDF 2627 kb) [file 40359_2017_196_MOESM1_ESM.pdf]

Assisting an Aboriginal or Torres Strait Islander person with gambling problems

Introduction

Purpose of this research

The purpose of this project is to develop a set of guidelines for the public on how to support an Aboriginal or Torres Strait Islander person with gambling problems to recover. This project has received funding from the Australian Government Department of Health.

Your role

You have been selected as a panel member for this study because you are 18 years or over AND

- are a gambling counsellor or researcher who is informed about Aboriginal gambling AND
- Have a minimum of 2 years' experience in researching or treating gambling problems in Aboriginal people.

Your task is to rate the statements presented in this questionnaire according to how important you believe they are for providing support to an Aboriginal or Torres Strait Islander person with gambling problems recover.

How this questionnaire was developed

The statements in this questionnaire were derived from information collected during a literature review of websites, books, and journal articles on how to assist anyone with gambling problems, and also how to specifically assist an Aboriginal or Torres Strait Islander person with gambling problems. This review examined any written information about how a member of the public can assist a person with gambling problems.

Some of the statements may seem contradictory or controversial; however, we have included them because they reflect the wide range of people's beliefs about the best ways to provide assistance to someone with gambling problems. It is important to note that we do not necessarily agree with these statements; we have included them because we do not believe that we should decide what the best practice is in this area. Rather, we have invited you to be a member of the expert panel to help develop a set of guidelines that reflect current expert opinion.

You will note that there is a place for you to add comments at the end of each section. This is so you can suggest any additional statements you think are important to giving assistance to an Aboriginal or Torres Strait Islander person with gambling problems. These statements will then go into the Round 2 survey to be rated by the expert panel.

Assisting an Aboriginal or Torres Strait Islander person with gambling problems

Instructions

Please complete the questionnaire by rating each statement ***according to how important you believe it is for inclusion in the guidelines*** for assisting an Aboriginal or Torres Strait Islander person with gambling problems. Please keep in mind that the guidelines will be used by the general public. The statements need to be rated according to their importance for someone ***without a counselling or clinical background*** assisting an Aboriginal or Torres Strait Islander person with gambling problems.

This questionnaire should take approximately 60 minutes to complete. You can complete the survey in two or more sittings. Your answers are saved when you click ‘Next’ at the bottom of a page. This marks your page and you can begin again at a later date on the next page. ***Please be aware that once you have logged on and started responding you must complete the questionnaire on the same computer.***

Assisting an Aboriginal or Torres Strait Islander person with gambling problems

Criteria for participating in this research

To participate in this research you must be 18 years or over AND:
- Be a gambling counsellor or researcher who is informed about Aboriginal gambling AND
- Have a minimum of 2 years’ experience in researching or treating gambling problems in Aboriginal or Torres Strait Islander people.

* 1. Do you meet these criteria?

- ◯ Yes, I meet these criteria.
- ◯ No, I do not meet these criteria.

If you answered "No" to question 1, please exit the survey. If you answered "Yes" please continue to the next page.

Assisting an Aboriginal or Torres Strait Islander person with gambling problems

Consent to participate

It is important for you to know that participation in this study is completely voluntary. You are not under any obligation to participate and you can withdraw at any time.

We would like to thank you for your time and effort and encourage you to provide us with feedback on this process.

Best Wishes,
The Mental Health First Aid Research Team

* 2. I understand that by submitting this survey I am giving my consent to participate in this study.

◯ Yes, I understand.

◯ I do not consent to participate in this study.

If you answered "I do not consent to participate in this study" to question 2, please exit the survey. If you answered "Yes" please continue to the next page.

Assisting an Aboriginal or Torres Strait Islander person with gambling problems

Overview of the survey

This survey is divided into the following section:

SECTION 1: Cultural considerations when assisting an Aboriginal person with gambling problems
SECTION 2: Warning signs of a gambling problem
SECTION 3: Awareness about gambling and gambling problems
SECTION 4: Awareness about gambling treatment and recovery from gambling problems
SECTION 5: Communication skills
SECTION 6: First aid actions

Assisting an Aboriginal or Torres Strait Islander person with gambling problems

Definitions used in this survey

Aboriginal in this questionnaire refers to Aboriginal or Torres Strait Islander people.

First aider refers to a concerned family member, friend, work colleague, or work supervisor who provides assistance to an Aboriginal person with gambling problems.

The Aboriginal person refers to the Aboriginal or Torres Strait Islander person with gambling problems or suspected gambling problems.

Gambling is the staking of money on uncertain events that are driven by chance.

Gambling problems are difficulties over time in limiting money or time spent on gambling, which leads to adverse consequences for the person, others, or for the community. This could include someone whose gambling problems are at a clinically diagnosable level.

Venue refers to a virtual or land-based location offering gambling or gaming activities with the chance to win money.

Gambling first aid is the assistance given to the Aboriginal person who is developing a gambling problem, experiencing a worsening of their gambling problem or experiencing a mental health crisis related to gambling. The assistance is given until appropriate professional help is received or until the crisis resolves.

Assisting an Aboriginal or Torres Strait Islander person with gambling problems

Information about you

* 3. Which best describes your area of expertise:

- ◯ I work for an Aboriginal Health or Gambling Service.
- ◯ I work for a general Gambling Service, but have experience in assisting Aboriginal people with gambling problems.
- ◯ I work for a general Health Service, but have experience in assisting Aboriginal people with gambling problems.
- ◯ I am a researcher experienced in Aboriginal Gambling.
- ◯ Other (please specify)

* 4. Do you identify as an Aboriginal or Torres Strait Islander Person?

- ◯ Aboriginal
- ◯ Torres Strait Islander
- ◯ Both Aboriginal and Torres Strait Islander
- ◯ Neither Aboriginal nor Torres Strait Islander

* 5. Apart from your professional experience, do you also have personal experience with gambling problems in yourself, your family or in your community network? (Mark as many as apply)

- ☐ Myself
- ☐ My family
- ☐ My broader community network
- ☐ None of the above
- ☐ I'd rather not say
- ☐ Other (please specify)

* 6. How old are you (in years)?

* 7. What is your gender?

- Female
- Male
- Other
- Do not wish to disclose

* 8. What organisation or business do you work for?

* 9. Where do you work?

City/Town

State/Territory

Assisting an Aboriginal or Torres Strait Islander person with gambling problems

SECTION 1: Cultural considerations when assisting an Aboriginal person with gambling problems

This section contains statements about what the first aider needs to know about Aboriginal culture when providing assistance to an Aboriginal person with gambling problems.

There are already guidelines on cultural considerations and communication techniques (click here to access these guidelines) for providing mental health first aid to an Aboriginal person who may be experiencing a range of mental health problems (access the full range of guidelines). We do not wish to replicate these guidelines. Rather, our aim is to develop guidelines on how to assist an Aboriginal person who is experiencing gambling problems.

Please keep our definitions in mind when completing the survey. You can access the definitions here.

Please rate how important (from ‘essential’ to ‘should not be included’) you think it is that each statement be included in the guidelines.

* 10. The first aider should know that Aboriginal people understand mental health within a broad context of health and wellbeing, which includes concepts of social, spiritual and emotional functioning.

- ◯ Essential
- ◯ Important
- ◯ Don't know/Depends
- ◯ Unimportant
- ◯ Should not be included

* 11. The first aider should be aware that cultural identity and connection to country is a crucial element of everyday life for Aboriginal people.

- ◯ Essential
- ◯ Important
- ◯ Don't know/Depends
- ◯ Unimportant
- ◯ Should not be included

* 12. The first aider should be aware that an Aboriginal person’s sense of cultural distinctiveness is linked with their spiritual, emotional and social health and well-being.

- Essential
- Important
- Don't know/Depends
- Unimportant
- Should not be included

* 13. Although these guidelines are being produced for use nationally, it is recognised that Aboriginal culture is not homogenous and first aiders will need to be aware of the cultural considerations specific to the community in which they are assisting. Therefore the first aider should:

	Essential	Important	Don't know/Depends	Unimportant	Should not be included
Learn about the way the Aboriginal person's community views gambling, e.g. gambling is not considered a problem and is therefore not discussed or addressed, money is for spending, tomorrow is a new day with new opportunities.	○	○	○	○	○
Learn about the way the individual Aboriginal person views gambling.	○	○	○	○	○

* 14. The first aider should know that within some Aboriginal communities, gambling problems are seen as 'just the way the person is' rather than as a treatable health condition.

- Essential
- Important
- Don't know/Depends
- Unimportant
- Should not be included

SECTION 2: Warning signs - behavioural

This section contains statements about the warning signs of gambling problems.

Please keep our definitions in mind when completing the survey. You can access the definitions here.

Please rate how important (from ‘essential’ to ‘should not be included’) you think it is that each statement be included in the guidelines.

* 15. The first aider should be aware that the following behavioural signs indicate that an Aboriginal person may have gambling problems:

	Essential	Important	Don't know/Depends	Unimportant	Should not be included
The person frequently thinks and talks about gambling.	○	○	○	○	○
The person frequently uses gambling terms in everyday conversation, e.g. bet, favourite.	○	○	○	○	○
The person checks sports scores or racing results frequently, e.g. online, mobile device.	○	○	○	○	○
The person gambles rather than doing things they previously enjoyed.	○	○	○	○	○
The person expresses a strong desire or craving to gamble.	○	○	○	○	○
The person reports dreaming about gambling.	○	○	○	○	○
The person gambles almost every day.	○	○	○	○	○
The person gambles every day of the week.	○	○	○	○	○
The person complains of boredom when they are not gambling.	○	○	○	○	○
The person is restless when they are not gambling.	○	○	○	○	○

	Essential	Important	Don't know/Depends	Unimportant	Should not be included
The person expresses guilt about their gambling.	○	○	○	○	○
The person is evasive or defensive when questioned about missed events or responsibilities.	○	○	○	○	○
The person becomes defensive or angry when asked about their gambling.	○	○	○	○	○
The person blames others for their gambling or its consequences.	○	○	○	○	○
The person's family finds evidence of regular gambling, e.g. gambling receipts, Internet browser history, bank statements.	○	○	○	○	○
The person neglects the basic care of their children.	○	○	○	○	○
The person breaks promises to their children about buying them things or spending time with them due to gambling.	○	○	○	○	○
The person causes arguments so they can leave home and gamble.	○	○	○	○	○
The person lies to cover up gambling.	○	○	○	○	○
The person increases the number or range of venues they gamble in.	○	○	○	○	○
The person increases the time they spend gambling.	○	○	○	○	○
The person is evasive about gambling losses.	○	○	○	○	○

	Essential	Important	Don't know/Depends	Unimportant	Should not be included
The person expresses thoughts about gambling that are not realistic, e.g. "It must be my turn for a large win."					
The person boasts about gambling wins.					
The person continues to gamble despite promising to stop.					
The person has tried unsuccessfully to control, cut back or stop gambling.					
The person has a pattern of gambling for longer than intended.					
The person is away from home for unexplained periods of time.					
The person lies to fund gambling.					
The person commits illegal acts to fund gambling, e.g. embezzlement, fraud.					
The person steals from family or friends to fund gambling.					
The person has legal problems related to gambling.					
Before gambling, the person expresses a fear that they may miss an opportunity to win, e.g. "If I don't gamble today my lucky numbers will come up and I will miss out."					
Before gambling, the person is over-confident or expresses fantasies about winning.					

	Essential	Important	Don't know/Depends	Unimportant	Should not be included
Before gambling, the person expresses excitement in anticipation of gambling.	○	○	○	○	○
After winning, the person expresses relief.	○	○	○	○	○
After winning, the person expresses a conviction that the win was the result of their skill.	○	○	○	○	○
After winning, the person appears to have an elated mood.	○	○	○	○	○
The person celebrates their wins by gambling more.	○	○	○	○	○
After losing, the person expresses fear of others finding out.	○	○	○	○	○
After losing, the person expresses worry over where they will get money to cover living expenses.	○	○	○	○	○
After losing, the person expresses anger towards themselves.	○	○	○	○	○
After losing, the person uses alcohol to forget about gambling problems.	○	○	○	○	○
After losing, the person expresses anger towards family or friends.	○	○	○	○	○
After losing, the person expresses hopelessness.	○	○	○	○	○
After losing, the person engages in risky behaviour.	○	○	○	○	○
The person reports that they cannot recall periods when they were gambling.	○	○	○	○	○

	Essential	Important	Don't know/Depends	Unimportant	Should not be included
The person has a pattern of returning to gambling in order to recover losses.	○	○	○	○	○

16. Please provide any additional behavioural signs:

Assisting an Aboriginal or Torres Strait Islander person with gambling problems

SECTION 2: Warning signs - while gambling

This section contains statements about the warning signs of gambling problems.

Please keep our definitions in mind when completing the survey. You can access the definitions here.

Please rate how important (from 'essential' to 'should not be included') you think it is that each statement be included in the guidelines.

* 17. The first aider should be aware that the following signs indicate that an Aboriginal person may have gambling problems:

	Essential	Important	Don't know/Depends	Unimportant	Should not be included
The person gambles for three or more hours without a break of at least 15 minutes.	○	○	○	○	○
The person gambles for five or more hours without a break of at least 15 minutes.	○	○	○	○	○
The person focuses so intensely on gambling that they don't react to what is going on around them.	○	○	○	○	○
While gambling, the person avoids contact with others or communicates very little with anyone else.	○	○	○	○	○
The person bets above the minimum per spin most of the time.	○	○	○	○	○
The person has a significant change in their gambling expenditure pattern, e.g. sudden increases in size of bets.	○	○	○	○	○
The person stays on to gamble after friends leave the venue.	○	○	○	○	○

	Essential	Important	Don't know/Depends	Unimportant	Should not be included
The person stops gambling only when the venue is closing.	◯	◯	◯	◯	◯
The person finds it difficult to stop gambling at closing time.	◯	◯	◯	◯	◯
The person regularly starts gambling as soon as the venue is open.	◯	◯	◯	◯	◯
The person asks venue staff to not let other people know that they are there.	◯	◯	◯	◯	◯
The person has friends or relatives call or arrive at the venue asking if they are still there.	◯	◯	◯	◯	◯
The person withdraws cash two or more times while at a gambling venue.	◯	◯	◯	◯	◯
The person borrows money from others while at the venue.	◯	◯	◯	◯	◯
The person asks for a loan or credit from the venue.	◯	◯	◯	◯	◯
The person gambles with large wins.	◯	◯	◯	◯	◯
The person leaves the venue to find money so that they can continue gambling.	◯	◯	◯	◯	◯
The person rummages around in purse or wallet for additional money to gamble.	◯	◯	◯	◯	◯
The person gambles until all the money they have with them is used up.	◯	◯	◯	◯	◯
The person is shaking while gambling.	◯	◯	◯	◯	◯
The person sweats a lot while gambling.	◯	◯	◯	◯	◯

	Essential	Important	Don't know/Depends	Unimportant	Should not be included
The person looks nervous or edgy, e.g. leg twitching, bites lip continuously.	○	○	○	○	○
The person displays anger, e.g. swears to themselves, grunts, kicks or strikes.	○	○	○	○	○
The person looks very sad or depressed after gambling.	○	○	○	○	○
The person cries after losing a lot of money.	○	○	○	○	○
The person sits with their head in their hands after losing.	○	○	○	○	○
The person blames venues or gaming machines for losing.	○	○	○	○	○
The person swears at or complains to staff about losing.	○	○	○	○	○
The person groans repeatedly while gambling.	○	○	○	○	○
The person shows significant changes in mood during a gambling session.	○	○	○	○	○
The person appears to avoid the cashier or appears evasive by only using cash machine.	○	○	○	○	○
The person gambles after having drunk a lot of alcohol.	○	○	○	○	○
The person plays the gaming machine very roughly and aggressively, e.g. with fists or slaps.	○	○	○	○	○
The person stands over other players while waiting for his or her favourite gaming machine.	○	○	○	○	○

	Essential	Important	Don't know/Depends	Unimportant	Should not be included
The person becomes angry if someone takes their favourite gaming machine or spot in the venue.	○	○	○	○	○
After winning on gaming machines, the person plays on quickly without stopping to listen to the music or jingle.	○	○	○	○	○
The person gambles on 2 or more gaming machines at once (where this is allowed).	○	○	○	○	○
The person plays faster than most (e.g. inserts large numbers of coins into the gaming machine very rapidly, presses the buttons very rapidly so that the spin rate is very fast).	○	○	○	○	○
The person performs ritualistic or superstitious routines while gambling, e.g. compulsively rubs belly of machine or screen while playing.	○	○	○	○	○
The person rushes from one gaming machine or gaming table to another.	○	○	○	○	○
The person is particular about reserving their machine while they take a break.	○	○	○	○	○
The person often gambles on their own.	○	○	○	○	○

18. Please provide any additional signs that are evident while gambling:

SECTION 2: Warning signs - while at work

This section contains statements about the warning signs of gambling problems.

Please keep our definitions in mind when completing the survey. You can access the definitions here.

Please rate how important (from ‘essential’ to ‘should not be included’) you think it is that each statement be included in the guidelines.

* 19. The first aider should be aware that the following signs indicate that an Aboriginal person may have gambling problems:

	Essential	Important	Don't know/Depends	Unimportant	Should not be included
The person gambles during work time.	○	○	○	○	○
The person repeatedly violates company gambling policy.	○	○	○	○	○
The person's ability to work or study has been negatively affected as a result of gambling.	○	○	○	○	○
The person is away from work for unexplained periods of time.	○	○	○	○	○
The person's use of company money seems suspicious or inappropriate.	○	○	○	○	○
The person plans holidays around gambling.	○	○	○	○	○
The person receives visits or phone calls from debt collectors while at work.	○	○	○	○	○
The person asks for advances on their pay.	○	○	○	○	○
The person borrows money from co-workers.	○	○	○	○	○
The person argues with co-workers over money owed.	○	○	○	○	○

	Essential	Important	Don't know/Depends	Unimportant	Should not be included
The person steals items from their company to resell.	○	○	○	○	○
The person sells items at work (either personal or stolen items).	○	○	○	○	○
The person arranges to have personal financial statements sent to work rather than home.	○	○	○	○	○

20. Please provide any additional signs that are evident while at work:

Assisting an Aboriginal or Torres Strait Islander person with gambling problems

SECTION 2: Warning signs - financial

This section contains statements about the warning signs of gambling problems.

Please keep our definitions in mind when completing the survey. You can access the definitions here.

Please rate how important (from 'essential' to 'should not be included') you think it is that each statement be included in the guidelines.

* 21. The first aider should be aware that the following financial signs indicate that an Aboriginal person may have gambling problems:

	Essential	Important	Don't know/Depends	Unimportant	Should not be included
The family believes they can't trust the person with money.	○	○	○	○	○
Valuables disappear (and may reappear) without explanation.	○	○	○	○	○
The person makes promises to pay back family and friends but never does so.	○	○	○	○	○
The person is bad-tempered about money.	○	○	○	○	○
The person hides financial statements.	○	○	○	○	○
The person is secretive about money.	○	○	○	○	○
The person cannot explain missing amounts of money from the house or bank accounts.	○	○	○	○	○
The person alternates between having no money and having a lot of money.	○	○	○	○	○
The person constantly swaps money from one account to another.	○	○	○	○	○
The person has numerous personal loans.	○	○	○	○	○

	Essential	Important	Don't know/Depends	Unimportant	Should not be included
The family finds hidden and unexplained money, e.g. money in a separate bank account, cash hidden in a drawer.	○	○	○	○	○
The person's family has to hide money from the person in order to cover living expenses.	○	○	○	○	○
The person or their family has experienced financial hardship as a result of gambling.	○	○	○	○	○
The person is consistently late in paying bills or misses payments entirely.	○	○	○	○	○
The person's essential services are frequently being disconnected, e.g. electricity, phone.	○	○	○	○	○
The person complains about mounting debts.	○	○	○	○	○
The person has a history of defaulting on payments.	○	○	○	○	○
The person or their family is frequently bothered by debt collectors.	○	○	○	○	○
The person owes money to a loan shark.	○	○	○	○	○
The person takes on extra jobs or works for overtime pay, but has no money to show for it.	○	○	○	○	○
The person cashes in investments or other assets early.	○	○	○	○	○
The person is frequently short of money.	○	○	○	○	○
The person has a pattern of unexplained loss of money.	○	○	○	○	○

	Essential	Important	Don't know/Depends	Unimportant	Should not be included
The person has a history of reporting that their money has been stolen or lost.	◯	◯	◯	◯	◯
The person increases their usage of or acquires additional credit cards.	◯	◯	◯	◯	◯
The person borrows money to gamble or to pay gambling debts.	◯	◯	◯	◯	◯
The person has a pattern of spending all available funds in an episode of gambling.	◯	◯	◯	◯	◯
Over time, the person increases the amount of money spent on gambling.	◯	◯	◯	◯	◯
The person believes that gambling will solve their financial difficulties or bring material wealth.	◯	◯	◯	◯	◯
The person does not want to spend money on anything but gambling.	◯	◯	◯	◯	◯
The person is aware of all the ATM/money machines close to the venue.	◯	◯	◯	◯	◯

22. Please provide any additional financial signs:

Assisting an Aboriginal or Torres Strait Islander person with gambling problems

SECTION 2: Warning signs - mental and physical health

This section contains statements about the warning signs of gambling problems.

Please keep our definitions in mind when completing the survey. You can access the definitions here.

Please rate how important (from 'essential' to 'should not be included') you think it is that each statement be included in the guidelines.

* 23. The first aider should be aware that the following mental and physical health signs indicate that an Aboriginal person may have gambling problems:

	Essential	Important	Don't know/Depends	Unimportant	Should not be included
The person does not look after their health as a result of their gambling, e.g. does not take medication or eat a healthy diet.	○	○	○	○	○
The person does not look after personal hygiene due to gambling problems.	○	○	○	○	○
The person has frequent unexplained mood swings.	○	○	○	○	○
The person has experienced negative emotions as a result of gambling, e.g. sadness, anxiety, stress, anger.	○	○	○	○	○
The person's family has experienced negative emotions as a result of gambling, e.g. sadness, anxiety, stress, anger.	○	○	○	○	○
The person's mental health has been negatively affected as a result of gambling.	○	○	○	○	○
The mental health of the person's family has been negatively affected as a result of gambling.	○	○	○	○	○

	Essential	Important	Don't know/Depends	Unimportant	Should not be included
The person experiences remorse or feels depressed after gambling.	○	○	○	○	○
The person's self-esteem is tied to their gambling wins and losses.	○	○	○	○	○
The person gambles to escape problems.	○	○	○	○	○
The person's physical health has been negatively affected as a result of gambling.	○	○	○	○	○
The physical health of the person's family has been negatively affected as a result of gambling.	○	○	○	○	○

24. Please provide any additional mental or physical health signs:

SECTION 2: Warning signs - social and cultural

This section contains statements about the warning signs of gambling problems.

Please keep our definitions in mind when completing the survey. You can access the definitions here.

Please rate how important (from ‘essential’ to ‘should not be included’) you think it is that each statement be included in the guidelines.

* 25. The first aider should be aware that the following social and cultural signs indicate that an Aboriginal person may have gambling problems:

	Essential	Important	Don't know/Depends	Unimportant	Should not be included
The person's relationships have been negatively affected as a result of gambling.	○	○	○	○	○
The person's or their family's social life has been negatively affected as a result of gambling.	○	○	○	○	○
The person's partner is threatening to leave or break up the family due to the gambling.	○	○	○	○	○
The person has conflicts with others about money.	○	○	○	○	○
The person is criticised by others for their gambling.	○	○	○	○	○
The person's reputation has suffered due to gambling.	○	○	○	○	○
The person disappears from social events where gambling is also available, in order to gamble.	○	○	○	○	○
The person is unable to be emotionally present or involved in social situations because they are pre-occupied with gambling.	○	○	○	○	○

	Essential	Important	Don't know/Depends	Unimportant	Should not be included
The person isolates themselves because of gambling.	◯	◯	◯	◯	◯
The person exploits others to pay for their gambling.	◯	◯	◯	◯	◯
The person does not meet their cultural obligations due to their gambling, e.g. Elders and role models neglecting to pass down traditional knowledge, lack of loyalty and respect to their culture.	◯	◯	◯	◯	◯

26. Please provide any additional social or cultural signs:

* 27. The first aider should give the Aboriginal person a list of the signs of gambling problems and ask the Aboriginal person to consider if any of the signs apply to them.

◯ Essential

◯ Important

◯ Don't know/Depends

◯ Unimportant

◯ Should not be included

Assisting an Aboriginal or Torres Strait Islander person with gambling problems

SECTION 3: Awareness about gambling and gambling problems

This section contains statements about what the first aider needs to know about gambling and gambling problems when providing assistance to an Aboriginal person with gambling problems.

Please keep our definitions in mind when completing the survey. You can access the definitions here.

Please rate how important (from ‘essential’ to ‘should not be included’) you think it is that each statement be included in the guidelines.

Prevalence and risk factor for gambling and gambling problems

* 28. The first aider should be aware that gambling problems are more common in Aboriginal people than in the general population.

- Essential
- Important
- Don't know/Depends
- Unimportant
- Should not be included

* 29. The first aider should know that gambling problems are more common in rural Aboriginal people than in urban/non-rural Aboriginal people.

- Essential
- Important
- Don't know/Depends
- Unimportant
- Should not be included

* 30. The first aider should be aware of the risk factors for problem gambling.

- Essential
- Important
- Don't know/Depends
- Unimportant
- Should not be included

* 31. The first aider should be aware of the specific risk factors for problem gambling in Aboriginal people

- Essential
- Important
- Don't know/Depends
- Unimportant
- Should not be included

32. Please provide any additional items or comments related to this section.

SECTION 3: Awareness about gambling and gambling problems (cont.)

This section contains statements about what the first aider needs to know about gambling and gambling problems when providing assistance to an Aboriginal person with gambling problems.

Please keep our definitions in mind when completing the survey. You can access the definitions here.

Please rate how important (from 'essential' to 'should not be included') you think it is that each statement be included in the guidelines.

Motivations for gambling

* 33. The first aider should know about the common motivations for gambling, e.g. to win money, or because it is fun, exciting or social.

- ◯ Essential
- ◯ Important
- ◯ Don't know/Depends
- ◯ Unimportant
- ◯ Should not be included

* 34. The first aider should know about the common motivations for gambling in people with gambling problems, e.g. chasing losses, escaping negative emotions, building self-esteem.

- ◯ Essential
- ◯ Important
- ◯ Don't know/Depends
- ◯ Unimportant
- ◯ Should not be included

* 35. The first aider should know that while the motivations for gambling may be the same for Aboriginal and non-Aboriginal people, the motivation to gamble may be stronger in Aboriginal people due to the specific pressures some Aboriginal people encounter, e.g. need to get out of house due to overcrowding or other pressures at home, social and economic disadvantage, welfare dependence.

- Essential
- Important
- Don't know/Depends
- Unimportant
- Should not be included

* 36. The first aider should be aware that the Aboriginal person may see gambling as the only way to make up for losses.

- Essential
- Important
- Don't know/Depends
- Unimportant
- Should not be included

* 37. The first aider should know that the Aboriginal person may feel the need to gamble with increasing amounts of money as a way to achieve desired feelings of excitement.

- Essential
- Important
- Don't know/Depends
- Unimportant
- Should not be included

* 38. The first aider should be aware of the various functions of card games for Aboriginal communities, e.g. to relieve boredom, accumulating large sums of money, redistributing money within the community.

- Essential
- Important
- Don't know/Depends
- Unimportant
- Should not be included

* 39. The first aiders should be aware that card games are no longer performing the redistributive function because the winnings are often being spent outside of the community.

- Essential
- Important
- Don't know/Depends
- Unimportant
- Should not be included

* 40. The first aider should be aware of the positive effects of gambling in Aboriginal communities, e.g. sharing common interests, building or maintaining social networks, reducing social isolation.

- Essential
- Important
- Don't know/Depends
- Unimportant
- Should not be included

* 41. The first aider should be aware that the social benefits of gambling are declining with the increase in use of commercial gambling, e.g. poker machines.

- Essential
- Important
- Don't know/Depends
- Unimportant
- Should not be included

42. Please provide any additional items or comments related to this section.

SECTION 3: Awareness about gambling and gambling problems (cont.)

This section contains statements about what the first aider needs to know about gambling and gambling problems when providing assistance to an Aboriginal person with gambling problems.

Please keep our definitions in mind when completing the survey. You can access the definitions here.

Please rate how important (from 'essential' to 'should not be included') you think it is that each statement be included in the guidelines.

Awareness about gambling problems

* 43. The first aider should learn all they can about gambling problems by reading about them.

- ◯ Essential
- ◯ Important
- ◯ Don't know/Depends
- ◯ Unimportant
- ◯ Should not be included

* 44. The first aider should know that some types of gambling can cause more problems than others (e.g. gaming machines), however any form of gambling can become a problem.

- ◯ Essential
- ◯ Important
- ◯ Don't know/Depends
- ◯ Unimportant
- ◯ Should not be included

* 45. The first aider should know that gambling problems are mental health problems.

- ◯ Essential
- ◯ Important
- ◯ Don't know/Depends
- ◯ Unimportant
- ◯ Should not be included

* 46. The first aider should be aware that people with gambling problems are likely to have other mental health problems, e.g. depression, anxiety, alcohol or other drug use problems.

- ◯ Essential
- ◯ Important
- ◯ Don't know/Depends
- ◯ Unimportant
- ◯ Should not be included

* 47. The first aider should be aware that there is an increased risk of violence in families affected by gambling problems.

- ◯ Essential
- ◯ Important
- ◯ Don't know/Depends
- ◯ Unimportant
- ◯ Should not be included

* 48. The first aider should be aware that a person with gambling problems may not see them as a problem, even though it may be obvious to those around them.

- ◯ Essential
- ◯ Important
- ◯ Don't know/Depends
- ◯ Unimportant
- ◯ Should not be included

* 49. The first aider should be aware that a person with gambling problems may not see them as a problem, until they experience a crisis that they cannot solve themselves.

- ◯ Essential
- ◯ Important
- ◯ Don't know/Depends
- ◯ Unimportant
- ◯ Should not be included

* 50. The first aider should be aware that the Aboriginal person may transition in and out of gambling problems over time.

- Essential
- Important
- Don't know/Depends
- Unimportant
- Should not be included

* 51. The first aider should be aware that the Aboriginal person may go through cycles of awareness and denial that their gambling is a problem.

- Essential
- Important
- Don't know/Depends
- Unimportant
- Should not be included

* 52. The first aider should know that the Aboriginal person's family member or partner may not be aware or able to admit that the Aboriginal person has gambling problems.

- Essential
- Important
- Don't know/Depends
- Unimportant
- Should not be included

53. Please provide any additional items or comments related to this section.

Assisting an Aboriginal or Torres Strait Islander person with gambling problems

SECTION 4: Awareness about gambling treatment and recovery from gambling problems

This section contains statements about what the first aider needs to know about treatment and recovery when providing assistance to an Aboriginal person with gambling problems.

Please keep our definitions in mind when completing the survey. You can access the definitions here.

Please rate how important (from 'essential' to 'should not be included') you think it is that each statement be included in the guidelines.

Awareness about treatment and recovery

* 54. The first aider should understand that it is important to assist a person with gambling problems because of the possible significant consequences, e.g. relationship breakdown, criminal sanctions, loss of employment, suicide, poor physical and mental health.

- Essential
- Important
- Don't know/Depends
- Unimportant
- Should not be included

* 55. The first aider should know that gambling problems can be successfully treated.

- Essential
- Important
- Don't know/Depends
- Unimportant
- Should not be included

* 56. The first aider should know that the goals of treatment could be either abstinence from gambling or restricting gambling.

- Essential
- Important
- Don't know/Depends
- Unimportant
- Should not be included

* 57. The first aider should not assume that a gambling problem is a phase the Aboriginal person is likely to pass through.

- Essential
- Important
- Don't know/Depends
- Unimportant
- Should not be included

* 58. The first aider should be aware that recovery from gambling problems is a slow process.

- Essential
- Important
- Don't know/Depends
- Unimportant
- Should not be included

* 59. The first aider should know that support from family and friends can assist the Aboriginal person's recovery.

- Essential
- Important
- Don't know/Depends
- Unimportant
- Should not be included

* 60. The first aider should encourage the Aboriginal person to seek treatment with their partner or a family member.

- Essential
- Important
- Don't know/Depends
- Unimportant
- Should not be included

61. Please provide any additional items or comments related to this section.

Assisting an Aboriginal or Torres Strait Islander person with gambling problems

SECTION 4: Awareness about gambling treatment and recovery from gambling problems

This section contains statements about what the first aider needs to know about treatment and recovery when providing assistance to an Aboriginal person with gambling problems.

Please keep our definitions in mind when completing the survey. You can access the definitions here.

Please rate how important (from ‘essential’ to ‘should not be included’) you think it is that each statement be included in the guidelines.

Awareness about treatment and recovery (cont.)

* 62. The first aider should be aware that some Aboriginal people view gambling problems as spiritual problems.

- Essential
- Important
- Don't know/Depends
- Unimportant
- Should not be included

* 63. If the person sees their gambling problems as spiritual problems, the first aider should encourage the Aboriginal person to see a traditional healer or go to a healing program.

- Essential
- Important
- Don't know/Depends
- Unimportant
- Should not be included

* 64. The first aider should know the Stages of Change Model and its implications for assisting the Aboriginal person.

- Essential
- Important
- Don't know/Depends
- Unimportant
- Should not be included

* 65. The first aider should be aware that the Aboriginal person may have tried and failed repeatedly to control, cut back or stop gambling.

- Essential
- Important
- Don't know/Depends
- Unimportant
- Should not be included

* 66. The first aider should be familiar with the effective treatments available for gambling problems.

- Essential
- Important
- Don't know/Depends
- Unimportant
- Should not be included

* 67. The first aider should be aware that it is not necessary for the Aboriginal person's recovery for their friends and family to be aware of their gambling problems.

- Essential
- Important
- Don't know/Depends
- Unimportant
- Should not be included

* 68. The first aider should be aware that they are not personally responsible for 'fixing' the Aboriginal person's gambling problems.

- Essential
- Important
- Don't know/Depends
- Unimportant
- Should not be included

69. Please provide any additional items or comments related to this section.

Assisting an Aboriginal or Torres Strait Islander person with gambling problems

SECTION 4: Awareness about gambling treatment and recovery from gambling problems.

This section contains statements about what the first aider needs to know about treatment and recovery when providing assistance to an Aboriginal person with gambling problems.

Please keep our definitions in mind when completing the survey. You can access the definitions here.

Please rate how important (from 'essential' to 'should not be included') you think it is that each statement be included in the guidelines.

Awareness about treatment resources

* 70. The first aider should be aware of available local Aboriginal specific services, e.g. Aboriginal gambling liaison person, Aboriginal gambling services, healing programs.

- ◯ Essential
- ◯ Important
- ◯ Don't know/Depends
- ◯ Unimportant
- ◯ Should not be included

* 71. The first aider should be aware that the Aboriginal person may need to access various services, including medical services gambling counselling, legal services, mental health services, financial counselling, vocational rehabilitation or social assistance.

- ◯ Essential
- ◯ Important
- ◯ Don't know/Depends
- ◯ Unimportant
- ◯ Should not be included

* 72. The first aider should be aware of resources to assist the Aboriginal person with their gambling problems.

- Essential
- Important
- Don't know/Depends
- Unimportant
- Should not be included

* 73. The first aider should inform the Aboriginal person about available sources of assistance.

- Essential
- Important
- Don't know/Depends
- Unimportant
- Should not be included

* 74. The first aider should provide information about self-help resources and support groups.

- Essential
- Important
- Don't know/Depends
- Unimportant
- Should not be included

* 75. The first aider should be aware of resources that can assist the Aboriginal person to manage their financial difficulties.

- Essential
- Important
- Don't know/Depends
- Unimportant
- Should not be included

* 76. The first aider should be aware of the specific barriers to help-seeking that Aboriginal people experience, e.g. lack of knowledge about and confidence in gambling help services, lack of culturally appropriate help services.

- Essential
- Important
- Don't know/Depends
- Unimportant
- Should not be included

* 77. The first aider should be aware that the Aboriginal person may not be comfortable using Aboriginal-controlled health or gambling services because of concerns about confidentiality and shame.

- Essential
- Important
- Don't know/Depends
- Unimportant
- Should not be included

* 78. The first aider should be aware that an Aboriginal person may be reluctant to seek help from a non-Aboriginal gambling service.

- Essential
- Important
- Don't know/Depends
- Unimportant
- Should not be included

* 79. The first aider should work together with the Aboriginal person to identify the most appropriate supports for the Aboriginal person.

- Essential
- Important
- Don't know/Depends
- Unimportant
- Should not be included

* 80. The first aider should encourage the Aboriginal person to seek the type of assistance that is most appropriate for them.

- ◯ Essential
- ◯ Important
- ◯ Don't know/Depends
- ◯ Unimportant
- ◯ Should not be included

* 81. The first aider should be aware of any local mechanisms for excluding people with gambling problems from venues.

- ◯ Essential
- ◯ Important
- ◯ Don't know/Depends
- ◯ Unimportant
- ◯ Should not be included

* 82. If the first aider gives the Aboriginal person details about gambling services, they should call the Aboriginal person to follow-up and check if they contacted the service.

- ◯ Essential
- ◯ Important
- ◯ Don't know/Depends
- ◯ Unimportant
- ◯ Should not be included

83. Please provide any additional items or comments related to this section.

Assisting an Aboriginal or Torres Strait Islander person with gambling problems

SECTION 5: Communication skills

This section contains statements about what the first aider needs to know about communication when providing assistance to an Aboriginal person with gambling problems.

Please keep our definitions in mind when completing the survey. You can access the definitions here.

Please rate how important (from 'essential' to 'should not be included') you think it is that each statement be included in the guidelines.

Preparing to talk with the person

* 84. The first aider should be prepared for the full range of responses they may encounter when talking with the Aboriginal person about their gambling problems, e.g. relief, anger.

- ◯ Essential
- ◯ Important
- ◯ Don't know/Depends
- ◯ Unimportant
- ◯ Should not be included

* 85. The first aider should prepare for approaching the Aboriginal person about their gambling problems by speaking in advance to a professional who is knowledgeable about gambling problems.

- ◯ Essential
- ◯ Important
- ◯ Don't know/Depends
- ◯ Unimportant
- ◯ Should not be included

* 86. The first aider should prepare by having information about available help for gambling problems.

- ◯ Essential
- ◯ Important
- ◯ Don't know/Depends
- ◯ Unimportant
- ◯ Should not be included

* 87. The first aider should choose an appropriate place to talk, e.g. private, away from distractions and interruptions.

- Essential
- Important
- Don't know/Depends
- Unimportant
- Should not be included

* 88. The first aider should pick a time when both the first aider and the Aboriginal person are feeling well, rather than tired or upset.

- Essential
- Important
- Don't know/Depends
- Unimportant
- Should not be included

* 89. The first aider should choose a time when there is enough time to talk.

- Essential
- Important
- Don't know/Depends
- Unimportant
- Should not be included

* 90. The first aider should know that it may be difficult to find an ideal time to talk with the Aboriginal person, therefore they should just talk with the Aboriginal person rather than wait for the 'perfect' timing.

- Essential
- Important
- Don't know/Depends
- Unimportant
- Should not be included

* 91. The first aider should know that sometimes the best time to talk to the Aboriginal person is when they are in crisis following a gambling occasion.

- Essential
- Important
- Don't know/Depends
- Unimportant
- Should not be included

* 92. The first aider should not approach the Aboriginal person alone if there is a possibility that the Aboriginal person may become violent.

- Essential
- Important
- Don't know/Depends
- Unimportant
- Should not be included

* 93. If the Aboriginal person does not show up for an arranged meeting, the first aider should not take this personally, but rather schedule another time.

- Essential
- Important
- Don't know/Depends
- Unimportant
- Should not be included

94. Please provide any additional items or comments related to this section.

Assisting an Aboriginal or Torres Strait Islander person with gambling problems

SECTION 5: Communication skills (cont.)

This section contains statements about what the first aider needs to know about communication when providing assistance to an Aboriginal person with gambling problems.

Please keep our definitions in mind when completing the survey. You can access the definitions here.

Please rate how important (from 'essential' to 'should not be included') you think it is that each statement be included in the guidelines.

Talking with the person

* 95. When approaching the Aboriginal person to discuss their gambling problems, the first aider should:

	Essential	Important	Don't know/Depends	Unimportant	Should not be included
Try to talk about the gambling problems in a calm and rational manner.	○	○	○	○	○
State some positive things about the Aboriginal person and their relationship with the first aider.	○	○	○	○	○
Use 'I' statements rather than 'you' statements, e.g. "I feel worried when I don't know when you are coming home or how much money you will have spent," rather than "You upset me when you are late and have spent all our money."	○	○	○	○	○
Talk about what they have noticed, e.g. that the Aboriginal person spends a lot of time at gambling venues.	○	○	○	○	○
Focus on specific concerns about the Aboriginal person's behaviour and its impact.	○	○	○	○	○

	Essential	Important	Don't know/Depends	Unimportant	Should not be included
Focus on the impact of the Aboriginal person's gambling behaviour rather than on the Aboriginal person themselves as the problem.	○	○	○	○	○
Focus on what the Aboriginal person is going to do about their gambling problems.	○	○	○	○	○
Ask the Aboriginal person for their perspective and allow them to tell their story.	○	○	○	○	○
Validate the Aboriginal person's experience and feelings, e.g. "I understand that gambling is important to you."	○	○	○	○	○

96. Please provide any additional items or comments related to this section.

Assisting an Aboriginal or Torres Strait Islander person with gambling problems

SECTION 5: Communication skills (cont.)

This section contains statements about what the first aider needs to know about communication when providing assistance to an Aboriginal person with gambling problems.

Please keep our definitions in mind when completing the survey. You can access the definitions here.

Please rate how important (from 'essential' to 'should not be included') you think it is that each statement be included in the guidelines.

Talking with the person

* 97. The first aider should avoid:

	Essential	Important	Don't know/Depends	Unimportant	Should not be included
Lecturing the Aboriginal person about their gambling problems.	○	○	○	○	○
Interrogating the Aboriginal person about their gambling.	○	○	○	○	○
Trying to convince the Aboriginal person to quit gambling.	○	○	○	○	○
Trying to control the Aboriginal person by threatening, bribing, crying, or nagging.	○	○	○	○	○
Arguing with the Aboriginal person.	○	○	○	○	○
Verbally or physically attacking the Aboriginal person.	○	○	○	○	○

98. Please provide any additional items or comments related to this section.

Assisting an Aboriginal or Torres Strait Islander person with gambling problems

SECTION 5: Communication skills (cont.)

This section contains statements about what the first aider needs to know about communication when providing assistance to an Aboriginal person with gambling problems.

Please keep our definitions in mind when completing the survey. You can access the definitions here.

Please rate how important (from ‘essential’ to ‘should not be included’) you think it is that each statement be included in the guidelines.

Talking with the person (cont.)

* 99. The first aider should be aware the Aboriginal person may make promises that they are unable to keep.

- ◯ Essential
- ◯ Important
- ◯ Don't know/Depends
- ◯ Unimportant
- ◯ Should not be included

* 100. The first aider should be aware that the Aboriginal person may use denial, minimisation, rationalisation or blaming when approached by the first aider about their gambling problems.

- ◯ Essential
- ◯ Important
- ◯ Don't know/Depends
- ◯ Unimportant
- ◯ Should not be included

* 101. The first aider should be aware that the Aboriginal person may lie about their gambling problems.

- ◯ Essential
- ◯ Important
- ◯ Don't know/Depends
- ◯ Unimportant
- ◯ Should not be included

* 102. Rather than telling the Aboriginal person what to do, the first aider should make suggestions, e.g. "Would you be comfortable seeing a gambling counsellor" rather than "You should go see a gambling counsellor".

- Essential
- Important
- Don't know/Depends
- Unimportant
- Should not be included

* 103. The first aider should encourage the Aboriginal person to talk to others who have experienced gambling problems, as they may relate better.

- Essential
- Important
- Don't know/Depends
- Unimportant
- Should not be included

* 104. The first aider should be aware that a person with gambling problems may feel ashamed or embarrassed.

- Essential
- Important
- Don't know/Depends
- Unimportant
- Should not be included

* 105. The first aider should explain to the Aboriginal person that they will try not to be judgmental and ask the Aboriginal person to tell them if they are.

- Essential
- Important
- Don't know/Depends
- Unimportant
- Should not be included

* 106. When talking to the Aboriginal person about their gambling problems, the first aider should use empathy and compassion.

- ◯ Essential
- ◯ Important
- ◯ Don't know/Depends
- ◯ Unimportant
- ◯ Should not be included

* 107. The first aider should listen to the Aboriginal person without passing judgement.

- ◯ Essential
- ◯ Important
- ◯ Don't know/Depends
- ◯ Unimportant
- ◯ Should not be included

* 108. If the first aider has not experienced gambling problems themselves, they should tell the Aboriginal person that although they do not understand what the Aboriginal person is going through, they are available to support them.

- ◯ Essential
- ◯ Important
- ◯ Don't know/Depends
- ◯ Unimportant
- ◯ Should not be included

* 109. The first aider should give the Aboriginal person enough time to tell their story because this will help them to open up and trust the first aider.

- ◯ Essential
- ◯ Important
- ◯ Don't know/Depends
- ◯ Unimportant
- ◯ Should not be included

* 110. The first aider should keep private any discussions with the Aboriginal person.

- Essential
- Important
- Don't know/Depends
- Unimportant
- Should not be included

* 111. The first aider should keep private any discussions with the Aboriginal person, unless there is concern about the safety of the Aboriginal person or others.

- Essential
- Important
- Don't know/Depends
- Unimportant
- Should not be included

112. Please provide any additional items or comments related to this section.

SECTION 5: Communication skills (cont.)

This section contains statements about what the first aider needs to know about communication when providing assistance to an Aboriginal person with gambling problems.

Please keep our definitions in mind when completing the survey. You can access the definitions here.

Please rate how important (from ‘essential’ to ‘should not be included’) you think it is that each statement be included in the guidelines.

Dealing with difficulties when interacting with the person

* 113. If the first aider thinks that their ability to assist the Aboriginal person is impeded by any negative attitudes towards the Aboriginal person's gambling or gambling in general, they should suggest the Aboriginal person talks with someone else.

- ◯ Essential
- ◯ Important
- ◯ Don't know/Depends
- ◯ Unimportant
- ◯ Should not be included

* 114. The first aider should be aware the Aboriginal person may not want to talk to them about their gambling problems because of feelings of shame.

- ◯ Essential
- ◯ Important
- ◯ Don't know/Depends
- ◯ Unimportant
- ◯ Should not be included

* 115. If the Aboriginal person does not want to talk to the first aider about their gambling problems, the first aider should consider enlisting someone who has a good relationship with the Aboriginal person to talk with them.

- Essential
- Important
- Don't know/Depends
- Unimportant
- Should not be included

* 116. If the Aboriginal person does not want to talk to the first aider about their gambling problems, the first aider should tell them about services that are available to help them.

- Essential
- Important
- Don't know/Depends
- Unimportant
- Should not be included

* 117. If the Aboriginal person doesn't want to talk about the gambling problems right now, the first aider should let them know that they are available to talk when the person is ready.

- Essential
- Important
- Don't know/Depends
- Unimportant
- Should not be included

* 118. If the Aboriginal person tries to change the subject, the first aider should keep the conversation focused on the gambling problems.

- Essential
- Important
- Don't know/Depends
- Unimportant
- Should not be included

* 119. If the Aboriginal person continues to deny or rationalise the problems, the first aider should end the discussion and try again at another time.

- Essential
- Important
- Don't know/Depends
- Unimportant
- Should not be included

* 120. If the conversation ends without a resolution, the first aider should try to get agreement from the Aboriginal person about another time to continue the discussion.

- Essential
- Important
- Don't know/Depends
- Unimportant
- Should not be included

* 121. If the conversation becomes unproductive or aggressive, the first aider should end the discussion and try again at another time.

- Essential
- Important
- Don't know/Depends
- Unimportant
- Should not be included

122. Please provide any additional items or comments related to this section.

Assisting an Aboriginal or Torres Strait Islander person with gambling problems

SECTION 6: Gambling first aid actions

This section contains statements about what the first aider needs to do when providing assistance to an Aboriginal person with gambling problems.

Please keep our definitions in mind when completing the survey. You can access the definitions here.

Please rate how important (from ‘essential’ to ‘should not be included’) you think it is that each statement be included in the guidelines.

Encouraging help seeking

* 123. The first aider should talk with the Aboriginal person as soon as they suspect the Aboriginal person has gambling problems, as early intervention may reduce the negative impact.

- ◯ Essential
- ◯ Important
- ◯ Don't know/Depends
- ◯ Unimportant
- ◯ Should not be included

* 124. The first aider should encourage the Aboriginal person to seek professional help for their gambling problems.

- ◯ Essential
- ◯ Important
- ◯ Don't know/Depends
- ◯ Unimportant
- ◯ Should not be included

* 125. The first aider can encourage help-seeking by pointing out that:

	Essential	Important	Don't know/Depends	Unimportant	Should not be included
Many people with gambling problems have benefited from professional help.	○	○	○	○	○
Many people with gambling problems have benefited from support groups.	○	○	○	○	○
Many people with gambling problems have benefited from self-help strategies.	○	○	○	○	○
It is possible to recover from gambling problems.	○	○	○	○	○
Seeking help is not a sign of weakness.	○	○	○	○	○
Seeking help is a sensible thing to do when experiencing a problem, like seeing a doctor for a health problem.	○	○	○	○	○
The sooner the problem is addressed the easier it is to overcome.	○	○	○	○	○
Any professional help will be confidential.	○	○	○	○	○

* 126. The first aider should work with the Aboriginal person to agree on acceptable behaviours, e.g. talking to a professional, staying within agreed spending limits.

- ○ Essential
- ○ Important
- ○ Don't know/Depends
- ○ Unimportant
- ○ Should not be included

127. Please provide any additional items or comments related to this section.

SECTION 6: Gambling first aid actions (cont.)

This section contains statements about what the first aider needs to do when providing assistance to an Aboriginal person with gambling problems.

Please keep our definitions in mind when completing the survey. You can access the definitions here.

Please rate how important (from 'essential' to 'should not be included') you think it is that each statement be included in the guidelines.

Encouraging help seeking (cont.)

* 128. If the Aboriginal person asks, the first aider should go with the Aboriginal person to an appointment for professional help.

- Essential
- Important
- Don't know/Depends
- Unimportant
- Should not be included

* 129. If the Aboriginal person asks, the first aider should go with the Aboriginal person to an appointment for professional help, but should not be present during the consultation.

- Essential
- Important
- Don't know/Depends
- Unimportant
- Should not be included

* 130. If the Aboriginal person is reluctant to seek help, the first aider should:

	Essential	Important	Don't know/Depends	Unimportant	Should not be included
Ask if they want to go to gambling help services with their partner, a family member or another support person.	◯	◯	◯	◯	◯
Tell the Aboriginal person that most people react in a supportive way when gambling problems are disclosed.	◯	◯	◯	◯	◯
Suggest they see a financial counsellor.	◯	◯	◯	◯	◯

* 131. The first aider should be aware that Aboriginal people with gambling problems may be more likely to seek assistance for financial problems than for their gambling problems.

◯ Essential

◯ Important

◯ Don't know/Depends

◯ Unimportant

◯ Should not be included

* 132. The first aider should **not** attempt to force the Aboriginal person to seek professional help or attend a support group.

◯ Essential

◯ Important

◯ Don't know/Depends

◯ Unimportant

◯ Should not be included

133. Please provide any additional items or comments related to this section.

SECTION 6: Gambling first aid actions (cont.)

This section contains statements about what the first aider needs to do when providing assistance to an Aboriginal person with gambling problems.

Please keep our definitions in mind when completing the survey. You can access the definitions here.

Please rate how important (from 'essential' to 'should not be included') you think it is that each statement be included in the guidelines.

Supporting change

* 134. The first aider should be clear about what they are willing to help the Aboriginal person with and what behaviours they will tolerate, although these boundaries can be revisited over time.

- Essential
- Important
- Don't know/Depends
- Unimportant
- Should not be included

* 135. The first aider should encourage the Aboriginal person to use self-help strategies.

- Essential
- Important
- Don't know/Depends
- Unimportant
- Should not be included

* 136. The first aider should **know about** the common false beliefs that can lead to gambling problems, e.g. belief that they can beat the system or superstitions about luck.

- Essential
- Important
- Don't know/Depends
- Unimportant
- Should not be included

* 137. The first aider should **explain to the Aboriginal person** about the common false beliefs that can lead to gambling problems, e.g. belief that they can beat the system or superstitions about luck.

- ◯ Essential
- ◯ Important
- ◯ Don't know/Depends
- ◯ Unimportant
- ◯ Should not be included

* 138. The first aider should encourage the Aboriginal person to learn about the strategies that gambling providers use to keep people gambling and maximise profits, e.g. gaming machines are designed to keep people playing and spending money.

- ◯ Essential
- ◯ Important
- ◯ Don't know/Depends
- ◯ Unimportant
- ◯ Should not be included

* 139. The first aider should encourage the Aboriginal person to avoid going to gambling venues even if they are not going to gamble (e.g. going to a pub for a meal where gambling is available).

- ◯ Essential
- ◯ Important
- ◯ Don't know/Depends
- ◯ Unimportant
- ◯ Should not be included

* 140. The first aider should ask the Aboriginal person if there are any problems that have led them to increasing their gambling behaviours.

- ◯ Essential
- ◯ Important
- ◯ Don't know/Depends
- ◯ Unimportant
- ◯ Should not be included

141. Please provide any additional items or comments related to this section.

SECTION 6: Gambling first aid actions (cont.)

This section contains statements about what the first aider needs to do when providing assistance to an Aboriginal person with gambling problems.

Please keep our definitions in mind when completing the survey. You can access the definitions here.

Please rate how important (from 'essential' to 'should not be included') you think it is that each statement be included in the guidelines.

Supporting change (cont.)

* 142. The first aider should **not**:

	Essential	Important	Don't know/Depends	Unimportant	Should not be included
Give the Aboriginal person money.	◯	◯	◯	◯	◯
Give the Aboriginal person money, except to avoid severe consequences, e.g. legal sanctions.	◯	◯	◯	◯	◯
Pay the Aboriginal person's debt.	◯	◯	◯	◯	◯
Pay the Aboriginal person's debt, except to avoid severe consequences, e.g. legal sanctions.	◯	◯	◯	◯	◯
Cover the Aboriginal person's basic living expenses.	◯	◯	◯	◯	◯
Deny the Aboriginal person's basic needs, e.g. food or shelter.	◯	◯	◯	◯	◯
Take responsibility away from the Aboriginal person for managing their financial problems.	◯	◯	◯	◯	◯
Cover for the Aboriginal person's behaviours.	◯	◯	◯	◯	◯
Lie to cover for the Aboriginal person's behaviours.	◯	◯	◯	◯	◯
Deny to themselves or others that the Aboriginal person has a problem.	◯	◯	◯	◯	◯

143. The first aider should **not** (cont.)

	Essential	Important	Don't know/Depends	Unimportant	Should not be included
Expect the Aboriginal person to be rational about their gambling.	○	○	○	○	○
Expect the Aboriginal person to immediately control their gambling.	○	○	○	○	○
Accept blame for the Aboriginal person's gambling problems.	○	○	○	○	○
Justify the Aboriginal person's gambling, to themselves or others.	○	○	○	○	○
Minimise the Aboriginal person's gambling problems in order to avoid conflict.	○	○	○	○	○
Go gambling with the Aboriginal person	○	○	○	○	○
Drop off or pick up the Aboriginal person from gambling activities.	○	○	○	○	○
Tell the Aboriginal person to just stop gambling.	○	○	○	○	○
Use shame or guilt to force the Aboriginal person to change.	○	○	○	○	○

144. Please provide any additional items or comments related to this section.

SECTION 6: Gambling first aid actions (cont.)

This section contains statements about what the first aider needs to do when providing assistance to an Aboriginal person with gambling problems.

Please keep our definitions in mind when completing the survey. You can access the definitions here.

Please rate how important (from 'essential' to 'should not be included') you think it is that each statement be included in the guidelines.

Supporting change (cont.)

* 145. If the first aider pays the Aboriginal person's debts or expenses, they should make arrangements for the Aboriginal person to pay back the loan, even if it is a small amount each week.

- Essential
- Important
- Don't know/Depends
- Unimportant
- Should not be included

* 146. The first aider should know that an Elder or community member may feel obligated to give money to the Aboriginal person.

- Essential
- Important
- Don't know/Depends
- Unimportant
- Should not be included

* 147. The first aider should discourage people such as family and friends from taking responsibility for the consequences of the gambling problems.

- Essential
- Important
- Don't know/Depends
- Unimportant
- Should not be included

148. Please provide any additional items or comments related to this section.

SECTION 6: Gambling first aid actions (cont.)

This section contains statements about what the first aider needs to do when providing assistance to an Aboriginal person with gambling problems.

Please keep our definitions in mind when completing the survey. You can access the definitions here.

Please rate how important (from 'essential' to 'should not be included') you think it is that each statement be included in the guidelines.

If the person does not want to change

* 149. The first aider should be aware that the Aboriginal person may not want to change their gambling behaviours.

- Essential
- Important
- Don't know/Depends
- Unimportant
- Should not be included

* 150. The first aider should be aware that the Aboriginal person can only be assisted if they are ready to change their gambling behaviours.

- Essential
- Important
- Don't know/Depends
- Unimportant
- Should not be included

* 151. The first aider should still try to assist the person, even if they do not want to change their gambling.

- Essential
- Important
- Don't know/Depends
- Unimportant
- Should not be included

* 152. If the Aboriginal person does not want to change their gambling behaviours, the first aider should ask the Aboriginal person if gambling and its consequences are getting in the way of them living the life they want to live.

- Essential
- Important
- Don't know/Depends
- Unimportant
- Should not be included

* 153. If the Aboriginal person does not want to change their gambling behaviours, the first aider should **sensitively** ask the Aboriginal person if gambling and its consequences are getting in the way of them living the life they want to live.

- Essential
- Important
- Don't know/Depends
- Unimportant
- Should not be included

* 154. If the Aboriginal person is unwilling to seek professional assistance, the first aider should set limits around what behaviours they are willing and unwilling to accept from the Aboriginal person.

- Essential
- Important
- Don't know/Depends
- Unimportant
- Should not be included

* 155. The first aider should not use ultimatums.

- Essential
- Important
- Don't know/Depends
- Unimportant
- Should not be included

* 156. The first aider should not threaten consequences for the Aboriginal person's gambling behaviour that they are not prepared to carry out.

- Essential
- Important
- Don't know/Depends
- Unimportant
- Should not be included

* 157. The first aider should tell the Aboriginal person that they will be available to assist the Aboriginal person when they are ready to change their gambling behaviours.

- Essential
- Important
- Don't know/Depends
- Unimportant
- Should not be included

158. Please provide any additional items or comments related to this section.

SECTION 6: Gambling first aid actions (cont.)

This section contains statements about what the first aider needs to do when providing assistance to an Aboriginal person with gambling problems.

Please keep our definitions in mind when completing the survey. You can access the definitions here.

Please rate how important (from ‘essential’ to ‘should not be included’) you think it is that each statement be included in the guidelines.

Reducing the negative impact

* 159. *If the Aboriginal person decides to continue gambling*, the first aider should encourage them to reduce the negative impact of gambling by:

	Essential	Important	Don't know/Depends	Unimportant	Should not be included
Limiting time spent on gambling.	◯	◯	◯	◯	◯
Limiting money spent on gambling.	◯	◯	◯	◯	◯
Only gambling with money that the Aboriginal person can afford to lose.	◯	◯	◯	◯	◯
Restricting their gambling to activities where the Aboriginal person has greater control over their behaviour, e.g. paying cards because they have better control than when they play pokies.	◯	◯	◯	◯	◯
Balancing time spent on gambling with other activities.	◯	◯	◯	◯	◯
Keeping a record of gambling wins and losses.	◯	◯	◯	◯	◯
Eating before gambling.	◯	◯	◯	◯	◯
Stopping gambling after a win.	◯	◯	◯	◯	◯
Taking regular breaks while gambling.	◯	◯	◯	◯	◯
Gambling with someone who limits their gambling.	◯	◯	◯	◯	◯
Leaving bank cards or credit cards at home.	◯	◯	◯	◯	◯
Gambling only when all debts are paid off.	◯	◯	◯	◯	◯

* 160. *If the Aboriginal person decides to continue gambling*, the first aider should discourage them from gambling with friends or family who have gambling problems.

- Essential
- Important
- Don't know/Depends
- Unimportant
- Should not be included

* 161. *If the Aboriginal person decides to continue gambling*, the first aider should encourage them to reduce the negative impact of gambling by not:

	Essential	Important	Don't know/Depends	Unimportant	Should not be included
Gambling to earn money or pay debts.	◯	◯	◯	◯	◯
Trying to win back gambling losses.	◯	◯	◯	◯	◯
Using borrowed money to gamble.	◯	◯	◯	◯	◯
Using personal investments or savings to gamble.	◯	◯	◯	◯	◯
Taking cash or bank cards to access extra money while gambling.	◯	◯	◯	◯	◯
Gambling when their judgment is impaired by alcohol or other drugs.	◯	◯	◯	◯	◯
Gambling to escape from problems or feelings.	◯	◯	◯	◯	◯
Gambling when angry or upset.	◯	◯	◯	◯	◯
Gambling alone.	◯	◯	◯	◯	◯

162. Please provide any additional items or comments related to this section.

SECTION 6: Gambling first aid actions (cont.)

This section contains statements about what the first aider needs to do when providing assistance to an Aboriginal person with gambling problems.

Please keep our definitions in mind when completing the survey. You can access the definitions here.

Please rate how important (from 'essential' to 'should not be included') you think it is that each statement be included in the guidelines.

Assisting the person who wants to change

* 163. If the Aboriginal person decides to seek professional assistance, the first aider should offer to support them during this, e.g. talk with them about issues, skills and exercises that come out of therapy.

- ◯ Essential
- ◯ Important
- ◯ Don't know/Depends
- ◯ Unimportant
- ◯ Should not be included

* 164. If the Aboriginal person decides to use self-help strategies, the first aider should offer to support them during this.

- ◯ Essential
- ◯ Important
- ◯ Don't know/Depends
- ◯ Unimportant
- ◯ Should not be included

* 165. The first aider should assist the Aboriginal person to list the advantages and disadvantages of gambling.

- ◯ Essential
- ◯ Important
- ◯ Don't know/Depends
- ◯ Unimportant
- ◯ Should not be included

* 166. The first aider should encourage the Aboriginal person to write down, on a daily basis, the negative consequences of gambling.

- Essential
- Important
- Don't know/Depends
- Unimportant
- Should not be included

* 167. The first aider should encourage the Aboriginal person to write down, on a daily basis, the positive consequences of **not** gambling.

- Essential
- Important
- Don't know/Depends
- Unimportant
- Should not be included

* 168. The first aider should help the Aboriginal person make a list of strategies that can help them change their gambling behaviours.

- Essential
- Important
- Don't know/Depends
- Unimportant
- Should not be included

* 169. If the Aboriginal person is attempting to change their gambling behaviours, the first aider should focus on the future, rather than past mistakes.

- Essential
- Important
- Don't know/Depends
- Unimportant
- Should not be included

* 170. If the Aboriginal person is attempting to change their gambling behaviours, the first aider should note any positive behavioural changes and congratulate the Aboriginal person on these.

- Essential
- Important
- Don't know/Depends
- Unimportant
- Should not be included

* 171. If the Aboriginal person sets a budget and asks for help sticking to it, the first aider should support them in this.

- Essential
- Important
- Don't know/Depends
- Unimportant
- Should not be included

172. Please provide any additional items or comments related to this section.

SECTION 6: Gambling first aid actions (cont.)

This section contains statements about what the first aider needs to do when providing assistance to an Aboriginal person with gambling problems.

Please keep our definitions in mind when completing the survey. You can access the definitions here.

Please rate how important (from 'essential' to 'should not be included') you think it is that each statement be included in the guidelines.

Assisting the person who wants to change (cont.)

* 173. The first aider should be aware that the Aboriginal person who has stopped or reduced their gambling may experience a gap in their life that gambling used to fill, e.g. reduction in social activities.

- Essential
- Important
- Don't know/Depends
- Unimportant
- Should not be included

* 174. The first aider should suggest activities that they can do with the Aboriginal person that do not involve gambling, e.g. going to the movies or to a restaurant, reconnecting with family and friends.

- Essential
- Important
- Don't know/Depends
- Unimportant
- Should not be included

* 175. If the Aboriginal person is experiencing anxiety, anger, stress, depression or boredom, the first aider should provide social support, as these may be triggers for worsening of gambling problems.

- Essential
- Important
- Don't know/Depends
- Unimportant
- Should not be included

* 176. The first aider should discuss with the Aboriginal person possible strategies for handling gambling urges and encourage the Aboriginal person to use them.

- Essential
- Important
- Don't know/Depends
- Unimportant
- Should not be included

* 177. *<u>If asked by the Aboriginal person</u>*, the first aider should discuss with them possible strategies for handling gambling urges and encourage the Aboriginal person to use them.

- Essential
- Important
- Don't know/Depends
- Unimportant
- Should not be included

* 178. If the Aboriginal person gambles online, the first aider should encourage them to use software programs that block certain sites, restrict access time or that monitor and report all activity from a computer.

- Essential
- Important
- Don't know/Depends
- Unimportant
- Should not be included

* 179. The first aider should encourage the Aboriginal person to do the following:

	Essential	Important	Don't know/Depends	Unimportant	Should not be included
Accept that they will not win back past gambling losses.	◯	◯	◯	◯	◯
Reduce the amount of time and money spent on gambling.	◯	◯	◯	◯	◯
Avoid spending time with people who are associated with gambling.	◯	◯	◯	◯	◯
Seek support from family, extended family, kin, friends or Respected Aboriginal people to assist them to change their gambling behaviour.	◯	◯	◯	◯	◯
Share their successes in reducing their gambling reduction with supportive people.	◯	◯	◯	◯	◯
Share their successes in gambling lapses with supportive people.	◯	◯	◯	◯	◯

180. Please provide any additional items or comments related to this section.

SECTION 6: Gambling first aid actions (cont.)

This section contains statements about what the first aider needs to do when providing assistance to an Aboriginal person with gambling problems.

Please keep our definitions in mind when completing the survey. You can access the definitions here.

Please rate how important (from 'essential' to 'should not be included') you think it is that each statement be included in the guidelines.

Supporting the person through relapse

* 181. The first aider should be aware that although relapse is a problem, a relapse does not indicate that the Aboriginal person cannot recover.

◯ Essential
◯ Important
◯ Don't know/Depends
◯ Unimportant
◯ Should not be included

* 182. If the Aboriginal person has a relapse, the first aider should tell them that this is not a sign of long-term failure of recovery.

◯ Essential
◯ Important
◯ Don't know/Depends
◯ Unimportant
◯ Should not be included

* 183. The first aider should explain to the Aboriginal person that gambling problems took time to develop, so it may take them some time, and more than one attempt, to change their gambling behaviours.

◯ Essential
◯ Important
◯ Don't know/Depends
◯ Unimportant
◯ Should not be included

* 184. The first aider should continue to offer support, even if the Aboriginal person has a relapse.

- Essential
- Important
- Don't know/Depends
- Unimportant
- Should not be included

185. Please provide any additional items or comments related to this section.

SECTION 6: Gambling first aid actions (cont.)

This section contains statements about what the first aider needs to do when providing assistance to an Aboriginal person with gambling problems.

Please keep our definitions in mind when completing the survey. You can access the definitions here.

Please rate how important (from 'essential' to 'should not be included') you think it is that each statement be included in the guidelines.

Financial strategies

* 186. The first aider should know that some of the general strategies for limiting access to money may not work for an Aboriginal person, e.g. leaving money at home may be a problem in shared households because others may take the money.

- Essential
- Important
- Don't know/Depends
- Unimportant
- Should not be included

* 187. The first aider should talk over any suggested strategies with the Aboriginal person to help them determine if this will work for them.

- Essential
- Important
- Don't know/Depends
- Unimportant
- Should not be included

* 188. In order to limit access to money for gambling, the first aider should ask the Aboriginal person to consider the following:

	Essential	Important	Don't know/Depends	Unimportant	Should not be included
Allowing someone else to manage their finances, e.g. partner or other family member.	◯	◯	◯	◯	◯
Setting up accounts and loans so that they require a second signature.	◯	◯	◯	◯	◯
Receiving an allowance from a family member so that the majority of the money can be used for household expenses.	◯	◯	◯	◯	◯
Paying all critical household expenses before paying off gambling debts.	◯	◯	◯	◯	◯
Having a trusted relative or close friend take temporary control of the Aboriginal person's access to funds.	◯	◯	◯	◯	◯
Arranging that they have access to a limited amount of money each day that covers daily expenses, e.g. lunch, parking, coffee.	◯	◯	◯	◯	◯
Voluntarily using a basics card which cannot be used for gambling or cash withdrawals.	◯	◯	◯	◯	◯

* 189. The first aider should encourage the Aboriginal person to be transparent about finances with their partner or family, e.g. mutual access to bank and credit card records.

◯ Essential

◯ Important

◯ Don't know/Depends

◯ Unimportant

◯ Should not be included

* 190. If the first aider gives the Aboriginal person cash for a specific purpose, they should ask the Aboriginal person to provide a store receipt and return any change.

- Essential
- Important
- Don't know/Depends
- Unimportant
- Should not be included

* 191. The first aider should be aware that the Aboriginal person may experience a sense of relief if someone else takes control of their finances.

- Essential
- Important
- Don't know/Depends
- Unimportant
- Should not be included

* 192. If the Aboriginal person asks for money to help cover bills or debts, the first aider should refer them to a relief agency or financial counselling service, rather than giving them money.

- Essential
- Important
- Don't know/Depends
- Unimportant
- Should not be included

* 193. The first aider should know that if they choose to lend the Aboriginal person money, it is unlikely to be repaid.

- Essential
- Important
- Don't know/Depends
- Unimportant
- Should not be included

194. Please provide any additional items or comments related to this section.

Section 6: Gambling first aid actions (cont.)

This section contains statements about what the first aider needs to do when providing assistance to an Aboriginal person with gambling problems.

Please keep our definitions in mind when completing the survey. You can access the definitions here.

Please rate how important (from 'essential' to 'should not be included') you think it is that each statement be included in the guidelines.

Intervention

An intervention is when a group of people who care about the person meet with the person to point out the behaviours that are causing problems and ask them to stop. An intervention includes a discussion around the consequences for the person if they do not get help for their gambling problem.

* 195. The first aider should **not** organise an 'intervention'.

- Essential
- Important
- Don't know/Depends
- Unimportant
- Should not be included

* 196. The first aider should consider organising an intervention to encourage the Aboriginal person to get professional assistance for their gambling problems.

- Essential
- Important
- Don't know/Depends
- Unimportant
- Should not be included

* 197. If the first aider decides to arrange an intervention, they should:

	Essential	Important	Don't know/Depends	Unimportant	Should not be included
Consider engaging the assistance of a professional when organising an intervention.	○	○	○	○	○
Include family, extended family, kin, friends or Respected Aboriginal people who they know will be able to support the Aboriginal person in their recovery.	○	○	○	○	○
Plan the goals and content of the intervention.	○	○	○	○	○
Organise the intervention in a way that the Aboriginal person feels supported and cared for rather than punished or shamed.	○	○	○	○	○
Practice the intervention with someone who is trained in conducting interventions (if available).	○	○	○	○	○
Only list the behaviours that the first aider has observed, not what they have been told.	○	○	○	○	○

198. Please provide any additional items or comments related to this section.

Assisting an Aboriginal or Torres Strait Islander person with gambling problems

Section 6: Gambling first aid actions (cont.)

This section contains statements about what the first aider needs to do when providing assistance to an Aboriginal person with gambling problems.

Please keep our definitions in mind when completing the survey. You can access the definitions here.

Please rate how important (from 'essential' to 'should not be included') you think it is that each statement be included in the guidelines.

Concerns for safety

* 199. The first aider should be aware that suicidal thoughts and behaviours are more common in people with gambling problems.

- Essential
- Important
- Don't know/Depends
- Unimportant
- Should not be included

* 200. The first aider should be aware that the Aboriginal person may see suicide as a way to avoid difficult confrontations with loved ones or creditors.

- Essential
- Important
- Don't know/Depends
- Unimportant
- Should not be included

* 201. The first aider should be aware that the Aboriginal person may see suicide as a viable solution to financial problems due to life insurance payouts.

- Essential
- Important
- Don't know/Depends
- Unimportant
- Should not be included

* 202. The first aider should be aware of the Mental Health First Aid Guidelines for how to assist someone with suicidal thoughts or behaviours. (Click here to access these guidelines.)

- Essential
- Important
- Don't know/Depends
- Unimportant
- Should not be included

* 203. If the first aider is afraid that the Aboriginal person may harm themselves or someone else, the first aider should seek professional support before taking action to deal with the Aboriginal person's gambling problems.

- Essential
- Important
- Don't know/Depends
- Unimportant
- Should not be included

* 204. The first aider should act to protect any children who are being neglected as a result of the Aboriginal person's gambling.

- Essential
- Important
- Don't know/Depends
- Unimportant
- Should not be included

* 205. The first aider should act to protect family members who may be at risk due to the Aboriginal person's gambling.

- Essential
- Important
- Don't know/Depends
- Unimportant
- Should not be included

* 206. If the Aboriginal person is involved in illegal activities related to their gambling, the first aider should:

	Essential	Important	Don't know/Depends	Unimportant	Should not be included
Encourage the Aboriginal person to stop these activities.	○	○	○	○	○
Encourage the Aboriginal person to seek legal assistance.	○	○	○	○	○

* 207. The first aider should be aware that supporting a person with a gambling problem can be difficult and should know how to access support for themselves.

- Essential
- Important
- Don't know/Depends
- Unimportant
- Should not be included

208. Please provide any additional items or comments related to this section.

Assisting an Aboriginal or Torres Strait Islander person with gambling problems

Thank you.

That is the end of the Round 1 survey. Thank you for sharing your time and expertise with us.

If participating in this survey has caused you distress and you wish to talk to someone about this, you can contact Lifeline on 13 11 14.

By pressing the 'Done' button your responses will be registered with our survey software. Once all panel members have lodged their responses, we will collate the data and send you a report on the findings and the Round 2 survey.

Kind Regards,

The Mental Health First Aid Research Team

Introduction and Instructions

Purpose of this research
The purpose of this project is to develop a set of guidelines for the public on how to support an Aboriginal or Torres Strait Islander person with gambling problems. This project has received funding from the Australian Government Department of Health.

How this questionnaire was developed
The statements in this questionnaire were derived from the results of the Round 1 survey. You will note that each statement is marked as either a 'new' or 'rerate' item. New items were derived from the comments provided in the first survey. An item is rerated when 80-89% of the panel members rated it as 'essential' or 'important'.

Instructions
Please complete the questionnaire by rating each statement according to how important you believe it is for inclusion in the guidelines for helping a person with gambling problems. Please keep in mind that the guidelines will be used by the **general public**. The statements need to be rated according to their importance for someone **without a counselling or clinical background**.

This questionnaire should take approximately 45 minutes to complete. You can complete the survey in two or more sittings. Your answers are saved when you click 'Next' at the bottom of a page. This marks your page and you can begin again at a later date on the next page. Please be aware that once you have logged on and started responding you must complete the questionnaire on the same computer.

IMPORTANT NOTE:

There are already guidelines on cultural considerations and communication techniques (click here to access these guidelines) for providing mental health first aid to an Aboriginal person who may be experiencing a range of mental health problems (access the full range of guidelines). **We do not wish to replicate these guidelines**. Rather, our aim is to develop guidelines on how to assist an Aboriginal person who is experiencing gambling problems.

Structure of this survey
This survey is divided into the following section:
SECTION 1: Cultural considerations when assisting an Aboriginal person with gambling problems
SECTION 2: Warning signs of a gambling problem
SECTION 3: Awareness about gambling and gambling problems
SECTION 4: Awareness about gambling treatment and recovery from gambling problems
SECTION 5: Communication skills
SECTION 6: First aid actions

* 1. Please provide us with your email address. (This allows us to track who completes the Round 2 survey and is then eligible to complete Round 3.)

Definitions used in this survey

Aboriginal in this questionnaire refers to Aboriginal or Torres Strait Islander people.

First aider refers to a concerned family member, friend, work colleague, or work supervisor who provides assistance to an Aboriginal person with gambling problems.

The Aboriginal person refers to the Aboriginal or Torres Strait Islander person with gambling problems or suspected gambling problems.

Gambling is the staking of money on uncertain events that are driven by chance.

Gambling problems are difficulties over time in limiting money or time spent on gambling, which leads to adverse consequences for the person, others, or for the community. This could include someone whose gambling problems are at a clinically diagnosable level.

Venue refers to a virtual or land-based location offering gambling or gaming activities with the chance to win money.

Gambling first aid is the assistance given to the Aboriginal person who is developing a gambling problem, experiencing a worsening of their gambling problem or experiencing a mental health crisis related to gambling. The assistance is given until appropriate professional help is received or until the crisis resolves.

SECTION 1: Cultural considerations when assisting an Aboriginal person with gambling problems

This section contains statements about what the first aider needs to know about Aboriginal culture when providing assistance to an Aboriginal person with gambling problems.

There are already guidelines on cultural considerations and communication techniques (click here to access these guidelines) for providing mental health first aid to an Aboriginal person who may be experiencing a range of mental health problems (access the full range of guidelines). We do not wish to replicate these guidelines. Rather, our aim is to develop guidelines on how to assist an Aboriginal person who is experiencing gambling problems.

Please keep our definitions in mind when completing the survey. You can access the definitions here.

Please rate how important (from ‘essential’ to ‘should not be included’) you think it is that each statement be included in the guidelines.

* 2. The first aider should learn about the specific gambling-related problems in the Aboriginal person's community, e.g. card games, pokies, online gambling. (New)

- ◯ Essential
- ◯ Important
- ◯ Don't know/Depends
- ◯ Unimportant
- ◯ Should not be included

* 3. The first aider should know how the expectation of 'providing for family' may impact on gambling problems, e.g. other family members may provide for the person if they lose their money for rent or food. (New)

- Essential
- Important
- Don't know/Depends
- Unimportant
- Should not be included

* 4. The first aider should be aware that gambling problems are very sensitive and stigmatised in Aboriginal communities, particularly if the first aider is not Aboriginal. (New)

- Essential
- Important
- Don't know/Depends
- Unimportant
- Should not be included

SECTION 2: Warning signs - behavioural

This section contains statements about the warning signs of gambling problems.

Please keep our definitions in mind when completing the survey. You can access the definitions here.

Please rate how important (from 'essential' to 'should not be included') you think it is that each statement be included in the guidelines.

* 5. The first aider should give the Aboriginal person a list of the signs of gambling problems and ask the Aboriginal person to consider if any of the signs apply to them. (Rerate)

- Essential
- Important
- Don't know/Depends
- Unimportant
- Should not be included

* 6. The first aider should be aware that the following behavioural signs indicate that an Aboriginal person may have gambling problems:

	Essential	Important	Don't know/Depends	Unimportant	Should not be included
a. The person gambles almost every day. (Rerate)	◯	◯	◯	◯	◯
b. The person predominantly gambles on payday. (New)	◯	◯	◯	◯	◯
c. The person complains of boredom when they are not gambling. (Rerate)	◯	◯	◯	◯	◯
d. The person rationalises their gambling by stating they need to pay bills, meet financial obligations, etc. (New)	◯	◯	◯	◯	◯
e. The person is evasive or defensive when questioned about missed events or responsibilities. (Rerate)	◯	◯	◯	◯	◯
f. The person becomes defensive or angry when asked about their gambling. (Rerate)	◯	◯	◯	◯	◯
g. The person's family finds evidence of regular gambling, e.g. gambling receipts, Internet browser history, bank statements. (Rerate)	◯	◯	◯	◯	◯
h. The person moves from community gambling (e.g. cards) to commercial gambling (e.g. pokies). (New)	◯	◯	◯	◯	◯
i. Over time, the person increases the types of gambling they play, e.g. horses, pokies, keno, etc. (New)	◯	◯	◯	◯	◯
j. The person is evasive about gambling losses. (Rerate)	◯	◯	◯	◯	◯

	Essential	Important	Don't know/Depends	Unimportant	Should not be included
k. The person expresses thoughts about gambling that are not realistic, e.g. "It must be my turn for a large win." (Rerate)	◯	◯	◯	◯	◯
l. The person boasts about gambling wins. (Rerate)	◯	◯	◯	◯	◯
m. The person organises card games regularly. (New)	◯	◯	◯	◯	◯
n. Before gambling, the person expresses excitement in anticipation of gambling. (Rerate)	◯	◯	◯	◯	◯
o. After winning, the person expresses relief. (Rerate)	◯	◯	◯	◯	◯
p. After winning, the person expresses a conviction that the win was the result of their skill. (Rerate)	◯	◯	◯	◯	◯
q. After losing, the person expresses fear of others finding out. (Rerate)	◯	◯	◯	◯	◯
r. After losing, the person uses alcohol to forget about gambling problems. (Rerate)	◯	◯	◯	◯	◯
s. The person, who would normally drink alcohol or smoke, gambles without alcohol or cigarette consumption. (New)	◯	◯	◯	◯	◯
t. The person offers themselves sexually to other in exchange for money for gambling or to pay gambling debts. (New)	◯	◯	◯	◯	◯

SECTION 2: Warning signs - while gambling

This section contains statements about the warning signs of gambling problems.

Please keep our definitions in mind when completing the survey. You can access the definitions here.

Please rate how important (from ‘essential’ to ‘should not be included’) you think it is that each statement be included in the guidelines.

* 7. The first aider should be aware that the following signs indicate that an Aboriginal person may have gambling problems:

	Essential	Important	Don't know/Depends	Unimportant	Should not be included
a. The person gambles for three or more hours without a break of at least 15 minutes. (Rerate)	◯	◯	◯	◯	◯
b. The person gambles for five or more hours without a break of at least 15 minutes. (Rerate)	◯	◯	◯	◯	◯
c. The person focuses so intensely on gambling that they don't react to what is going on around them. (Rerate)	◯	◯	◯	◯	◯
d. While gambling, the person avoids contact with others or communicates very little with anyone else. (Rerate)	◯	◯	◯	◯	◯
e. The person stays on to gamble after friends leave the venue. (Rerate)	◯	◯	◯	◯	◯
f. The person stops gambling only when the venue is closing. (Rerate)	◯	◯	◯	◯	◯
g. The person finds it difficult to stop gambling at closing time. (Rerate)	◯	◯	◯	◯	◯
h. The person regularly starts gambling as soon as the venue is open. (Rerate)	◯	◯	◯	◯	◯

	Essential	Important	Don't know/Depends	Unimportant	Should not be included
i. The person asks venue staff to not let other people know that they are there. (Rerate)	○	○	○	○	○
j. The person has friends or relatives call or arrive at the venue asking if they are still there. (Rerate)	○	○	○	○	○
k. The person withdraws cash two or more times while at a gambling venue. (Rerate)	○	○	○	○	○
l. The person borrows money from others while at the venue. (Rerate)	○	○	○	○	○
m. The person asks for a loan or credit from the venue. (Rerate)	○	○	○	○	○
n. The person gambles with large wins. (Rerate)	○	○	○	○	○
o. The person gambles until all the money they have with them is used up. (Rerate)	○	○	○	○	○
p. The person looks nervous or edgy, e.g. leg twitching, bites lip continuously. (Rerate)	○	○	○	○	○
q. The person displays anger, e.g. swears to themselves, grunts, kicks or strikes. (Rerate)	○	○	○	○	○
r. The person looks very sad or depressed after gambling. (Rerate)	○	○	○	○	○
s. The person cries after losing a lot of money. (Rerate)	○	○	○	○	○
t. The person shows significant changes in mood during a gambling session. (Rerate)	○	○	○	○	○

	Essential	Important	Don't know/Depends	Unimportant	Should not be included
u. The person plays the gaming machine very roughly and aggressively, e.g. with fists or slaps. (Rerate)	○	○	○	○	○
v. The person becomes angry if someone takes their favourite gaming machine or spot in the venue. (Rerate)	○	○	○	○	○
w. The person gambles on 2 or more gaming machines at once (where this is allowed). (Rerate)	○	○	○	○	○
x. The person often gambles on their own. (Rerate)	○	○	○	○	○

SECTION 2: Warning signs - while at work

This section contains statements about the warning signs of gambling problems.

Please keep our definitions in mind when completing the survey. You can access the definitions here.

Please rate how important (from 'essential' to 'should not be included') you think it is that each statement be included in the guidelines.

* 8. The first aider should be aware that the following signs indicate that an Aboriginal person may have gambling problems:

	Essential	Important	Don't know/Depends	Unimportant	Should not be included
a. The person repeatedly violates company gambling policy. (Rerate)	○	○	○	○	○
b. The person's use of company money seems suspicious or inappropriate. (Rerate)	○	○	○	○	○

SECTION 2: Warning signs - financial

This section contains statements about the warning signs of gambling problems.

Please keep our definitions in mind when completing the survey. You can access the definitions here.

Please rate how important (from ‘essential’ to ‘should not be included’) you think it is that each statement be included in the guidelines.

* 9. The first aider should be aware that the following financial signs indicate that an Aboriginal person may have gambling problems:

	Essential	Important	Don't know/Depends	Unimportant	Should not be included
a. The family believes they can't trust the person with money. (Rerate)	○	○	○	○	○
b. Valuables disappear (and may reappear) without explanation. (Rerate)	○	○	○	○	○
c. The person alternates between having no money and having a lot of money. (Rerate)	○	○	○	○	○
d. The person's family has to hide money from the person in order to cover living expenses. (Rerate)	○	○	○	○	○
e. The person frequently asks for money to gamble or to pay gambling debt. (New)	○	○	○	○	○
f. The person has a history of reporting that their money has been stolen or lost. (Rerate)	○	○	○	○	○

SECTION 2: Warning signs - mental and physical health

This section contains statements about the warning signs of gambling problems.

Please keep our definitions in mind when completing the survey. You can access the definitions here.

Please rate how important (from ‘essential’ to ‘should not be included’) you think it is that each statement be included in the guidelines.

* 10. The first aider should be aware that the following mental and physical health signs indicate that an Aboriginal person may have gambling problems:

	Essential	Important	Don't know/Depends	Unimportant	Should not be included
a. The person does not look after personal hygiene due to gambling problems. (Rerate)	○	○	○	○	○
b. The person's self-esteem is tied to their gambling wins and losses. (Rerate)	○	○	○	○	○
c. The physical health of the person's family has been negatively affected as a result of gambling. (Rerate)	○	○	○	○	○

SECTION 2: Warning signs - social and cultural

This section contains statements about the warning signs of gambling problems.

Please keep our definitions in mind when completing the survey. You can access the definitions here.

Please rate how important (from ‘essential’ to ‘should not be included’) you think it is that each statement be included in the guidelines.

* 11. The first aider should be aware that the following social and cultural signs indicate that an Aboriginal person may have gambling problems:

	Essential	Important	Don't know/Depends	Unimportant	Should not be included
a. The person has conflicts with others about money. (Rerate)	○	○	○	○	○
b. The person is criticised by others for their gambling. (Rerate)	○	○	○	○	○
c. The person exploits others to pay for their gambling. (Rerate)	○	○	○	○	○

SECTION 3: Awareness about gambling and gambling problems

This section contains statements about what the first aider needs to know about gambling and gambling problems when providing assistance to an Aboriginal person with gambling problems.

Please keep our definitions in mind when completing the survey. You can access the definitions here.

Please rate how important (from ‘essential’ to ‘should not be included’) you think it is that each statement be included in the guidelines.

Prevalence and risk factor for gambling and gambling problems

* 12. The first aider should be aware that gambling problems are more common in Aboriginal people than in the general population. (Rerate)

- Essential
- Important
- Don't know/Depends
- Unimportant
- Should not be included

Motivations for gambling

* 13. The first aider should be aware that the Aboriginal person may see gambling as the only way to make up for losses. (Rerate)

- Essential
- Important
- Don't know/Depends
- Unimportant
- Should not be included

* 14. The first aiders should be aware that card games are no longer performing the redistributive function because the winnings are often being spent outside of the community. (Rerate)

- Essential
- Important
- Don't know/Depends
- Unimportant
- Should not be included

* 15. The first aider should be aware of the positive effects of gambling in Aboriginal communities, e.g. sharing common interests, building or maintaining social networks, reducing social isolation. (Rerate)

- Essential
- Important
- Don't know/Depends
- Unimportant
- Should not be included

* 16. The first aider should be aware that the social benefits of gambling are declining with the increase in use of commercial gambling, e.g. poker machines. (Rerate)

- Essential
- Important
- Don't know/Depends
- Unimportant
- Should not be included

Awareness about gambling problems

* 17. The first aider should try to learn more about gambling problems, e.g. ring a gambling help line, talking with others, reading information. (New)

- Essential
- Important
- Don't know/Depends
- Unimportant
- Should not be included

* 18. The first aider should know that some types of gambling can cause more problems than others (e.g. gaming machines), however any form of gambling can become a problem. (Rerate)

- Essential
- Important
- Don't know/Depends
- Unimportant
- Should not be included

* 19. The first aider should be aware that there is an increased risk of violence in families affected by gambling problems. (Rerate)

- Essential
- Important
- Don't know/Depends
- Unimportant
- Should not be included

SECTION 4: Awareness about gambling treatment and recovery from gambling problems

This section contains statements about what the first aider needs to know about treatment and recovery when providing assistance to an Aboriginal person with gambling problems.

Please keep our definitions in mind when completing the survey. You can access the definitions here.

Please rate how important (from 'essential' to 'should not be included') you think it is that each statement be included in the guidelines.

Awareness about treatment and recovery

* 20. The first aider should know that the goals of treatment could be either abstinence from gambling or restricting gambling. (Rerate)

- Essential
- Important
- Don't know/Depends
- Unimportant
- Should not be included

* 21. The first aider should not assume that a gambling problem is a phase the Aboriginal person is likely to pass through. (Rerate)

- Essential
- Important
- Don't know/Depends
- Unimportant
- Should not be included

* 22. The first aider should encourage the Aboriginal person to seek treatment with their partner or a family member. (Rerate)

- Essential
- Important
- Don't know/Depends
- Unimportant
- Should not be included

* 23. The first aider should know the Stages of Change Model and its implications for assisting the Aboriginal person. (Rerate)

- Essential
- Important
- Don't know/Depends
- Unimportant
- Should not be included

SECTION 4: Awareness about gambling treatment and recovery from gambling problems.

This section contains statements about what the first aider needs to know about treatment and recovery when providing assistance to an Aboriginal person with gambling problems.

Please keep our definitions in mind when completing the survey. You can access the definitions here.

Please rate how important (from ‘essential’ to ‘should not be included’) you think it is that each statement be included in the guidelines.

Awareness about treatment resources

* 24. The first aider should be aware of available local Aboriginal specific services, e.g. Aboriginal gambling liaison person, Aboriginal gambling services, healing programs. (Rerate)

- Essential
- Important
- Don't know/Depends
- Unimportant
- Should not be included

* 25. The first aider should be aware that the Aboriginal person may not be comfortable using Aboriginal-controlled health or gambling services because of concerns about confidentiality and shame. (Rerate)

- Essential
- Important
- Don't know/Depends
- Unimportant
- Should not be included

* 26. If there are no culturally competent gambling help services available, the first aider should work with the person to identify supportive family and community members who they can talk to about their gambling problems. (New)

- Essential
- Important
- Don't know/Depends
- Unimportant
- Should not be included

* 27. If there are no culturally competent gambling help services available, the first aider should work with the person to identify existing services in the community where they can access support, e.g. early childhood centres, women's centres, etc. (New)

- Essential
- Important
- Don't know/Depends
- Unimportant
- Should not be included

* 28. The first aider should offer to make an appointment for the person with a gambling help or other service. (New)

- Essential
- Important
- Don't know/Depends
- Unimportant
- Should not be included

* 29. The first aider should be aware of any local mechanisms for excluding people with gambling problems from venues. (Rerate)

- Essential
- Important
- Don't know/Depends
- Unimportant
- Should not be included

* 30. If the first aider gives the Aboriginal person details about gambling services, they should ask the person if they can follow-up to check if they contacted the service. (New)

- Essential
- Important
- Don't know/Depends
- Unimportant
- Should not be included

* 31. The first aider should provide information about self-help resources and support groups. (Rerate)

- Essential
- Important
- Don't know/Depends
- Unimportant
- Should not be included

SECTION 5: Communication skills

This section contains statements about what the first aider needs to know about communication when providing assistance to an Aboriginal person with gambling problems.

Please keep our definitions in mind when completing the survey. You can access the definitions here.

Please rate how important (from ‘essential’ to ‘should not be included’) you think it is that each statement be included in the guidelines.

Preparing to talk with the person

* 32. The first aider should know that it may be a long-term investment to support an Aboriginal person with gambling problems, particularly if the first aider is not Aboriginal. (New)

- Essential
- Important
- Don't know/Depends
- Unimportant
- Should not be included

* 33. The first aider should plan for how they will approach the Aboriginal person and gently introduce their concerns about the person's gambling. (New)

- Essential
- Important
- Don't know/Depends
- Unimportant
- Should not be included

* 34. The first aider should prepare by having information about available help for gambling problems. (Rerate)

- Essential
- Important
- Don't know/Depends
- Unimportant
- Should not be included

* 35. The first aider should pick a time when both the first aider and the Aboriginal person are feeling well, rather than tired or upset. (Rerate)

- Essential
- Important
- Don't know/Depends
- Unimportant
- Should not be included

* 36. If the Aboriginal person does not show up for an arranged meeting, the first aider should not take this personally, but rather schedule another time. (Rerate)

- Essential
- Important
- Don't know/Depends
- Unimportant
- Should not be included

Talking to the person

* 37. The first aider should have a yarn with the person, before talking about their concerns about the person's gambling. (New)

- Essential
- Important
- Don't know/Depends
- Unimportant
- Should not be included

* 38. The first aider should try to find some common ground for discussion, then gradually build up towards talking about the person's gambling. (New)

- Essential
- Important
- Don't know/Depends
- Unimportant
- Should not be included

* 39. In order to get the Aboriginal person to open up, the first aider should encourage them to yarn about other members of the community who have experienced gambling problems. (New)

- Essential
- Important
- Don't know/Depends
- Unimportant
- Should not be included

* 40. Rather than focusing on problems when talking to the person, the first aider should ask about stories about family and gambling. (New)

- Essential
- Important
- Don't know/Depends
- Unimportant
- Should not be included

SECTION 5: Communication skills (cont.)

This section contains statements about what the first aider needs to know about communication when providing assistance to an Aboriginal person with gambling problems.

Please keep our definitions in mind when completing the survey. You can access the definitions here.

Please rate how important (from 'essential' to 'should not be included') you think it is that each statement be included in the guidelines.

Talking with the person (cont.)

* 41. When approaching the Aboriginal person to discuss their gambling problems, the first aider should:

	Essential	Important	Don't know/Depends	Unimportant	Should not be included
a. Talk about what they have noticed, e.g. that the Aboriginal person spends a lot of time at gambling venues. (Rerate)	◯	◯	◯	◯	◯
b. Focus on what the Aboriginal person is going to do about their gambling problems. (Rerate)	◯	◯	◯	◯	◯
c. Validate the Aboriginal person's experience and feelings, e.g. "I understand that gambling is important to you." (Rerate)	◯	◯	◯	◯	◯
d. Ask the Aboriginal person if they would like to have family or friends present during their conversations. (New)	◯	◯	◯	◯	◯
e. As far as possible, let the Aboriginal person set the pace and style of the interaction. (New)	◯	◯	◯	◯	◯
f. Reassure the Aboriginal person that they care and want to help. (New)	◯	◯	◯	◯	◯

* 42. The first aider should avoid trying to convince the Aboriginal person to quit gambling. (Rerate)

◯ Essential

◯ Important

◯ Don't know/Depends

◯ Unimportant

◯ Should not be included

* 43. The first aider should be aware the Aboriginal person may make promises that they are unable to keep. (Rerate)

- Essential
- Important
- Don't know/Depends
- Unimportant
- Should not be included

* 44. The first aider should be aware that the Aboriginal person may lie about their gambling problems. (Rerate)

- Essential
- Important
- Don't know/Depends
- Unimportant
- Should not be included

* 45. The first aider should encourage the Aboriginal person to talk to others who have experienced gambling problems, as they may relate better. (Rerate)

- Essential
- Important
- Don't know/Depends
- Unimportant
- Should not be included

SECTION 5: Communication skills (cont.)

This section contains statements about what the first aider needs to know about communication when providing assistance to an Aboriginal person with gambling problems.

Please keep our definitions in mind when completing the survey. You can access the definitions here.

Please rate how important (from 'essential' to 'should not be included') you think it is that each statement be included in the guidelines.

Dealing with difficulties when interacting with the person

* 46. If the Aboriginal person does not want to talk to the first aider about their gambling problems, the first aider should consider enlisting someone who has a good relationship with the Aboriginal person to talk with them. (Rerate)

- Essential
- Important
- Don't know/Depends
- Unimportant
- Should not be included

* 47. If the Aboriginal person does not want to talk to the first aider about their gambling problems, the first aider should tell them about services that are available to help them. (Rerate)

- Essential
- Important
- Don't know/Depends
- Unimportant
- Should not be included

SECTION 6: Gambling first aid actions

This section contains statements about what the first aider needs to do when providing assistance to an Aboriginal person with gambling problems.

Please keep our definitions in mind when completing the survey. You can access the definitions here.

Please rate how important (from ‘essential’ to ‘should not be included’) you think it is that each statement be included in the guidelines.

Encouraging help seeking

* 48. The first aider should encourage the Aboriginal person to seek professional help for their gambling problems. (Rerate)

- Essential
- Important
- Don't know/Depends
- Unimportant
- Should not be included

* 49. The first aider can encourage help-seeking by pointing out that:

	Essential	Important	Don't know/Depends	Unimportant	Should not be included
a. Many people with gambling problems have benefited from support groups. (Rerate)	○	○	○	○	○
b. Many people with gambling problems have benefited from self-help strategies. (Rerate)	○	○	○	○	○
c. Seeking help is not a sign of weakness. (Rerate)	○	○	○	○	○
d. The sooner the problem is addressed the easier it is to overcome. (Rerate)	○	○	○	○	○

* 50. The first aider should be aware that Aboriginal people with gambling problems may be more likely to seek assistance for financial problems than for their gambling problems. (Rerate)

- ○ Essential
- ○ Important
- ○ Don't know/Depends
- ○ Unimportant
- ○ Should not be included

SECTION 6: Gambling first aid actions (cont.)

This section contains statements about what the first aider needs to do when providing assistance to an Aboriginal person with gambling problems.

Please keep our definitions in mind when completing the survey. You can access the definitions here.

Please rate how important (from ‘essential’ to ‘should not be included’) you think it is that each statement be included in the guidelines.

Supporting change

* 51. The first aider should be clear about what they are willing to help the Aboriginal person with and what behaviours they will tolerate, although these boundaries can be revisited over time. (Rerate)

- Essential
- Important
- Don't know/Depends
- Unimportant
- Should not be included

* 52. The first aider should encourage the Aboriginal person to use self-help strategies. (Rerate)

- Essential
- Important
- Don't know/Depends
- Unimportant
- Should not be included

* 53. The first aider should **know about** the common false beliefs that can lead to gambling problems, e.g. belief that they can beat the system or superstitions about luck. (Rerate)

- Essential
- Important
- Don't know/Depends
- Unimportant
- Should not be included

* 54. The first aider should encourage the Aboriginal person to learn about the strategies that gambling providers use to keep people gambling and maximise profits, e.g. gaming machines are designed to keep people playing and spending money. (Rerate)

- Essential
- Important
- Don't know/Depends
- Unimportant
- Should not be included

* 55. The first aider should:

	Essential	Important	Don't know/Depends	Unimportant	Should not be included
a. Not deny the Aboriginal person's basic needs, e.g. food or shelter. (Rerate)	◯	◯	◯	◯	◯
b. Not deny to themselves or others that the Aboriginal person has a problem. (Rerate)	◯	◯	◯	◯	◯
c. Not expect the Aboriginal person to immediately control their gambling. (Rerate)	◯	◯	◯	◯	◯
d. Not accept blame for the Aboriginal person's gambling problems. (Rerate)	◯	◯	◯	◯	◯
e. Not drop off or pick up the Aboriginal person from gambling activities. (Rerate)	◯	◯	◯	◯	◯
f. Not tell the Aboriginal person to just stop gambling. (Rerate)	◯	◯	◯	◯	◯
g. Not suggest alternatives to gambling that may become addictive or a problem behaviour, e.g. drinking alcohol. (New)	◯	◯	◯	◯	◯

SECTION 6: Gambling first aid actions (cont.)

This section contains statements about what the first aider needs to do when providing assistance to an Aboriginal person with gambling problems.

Please keep our definitions in mind when completing the survey. You can access the definitions here.

Please rate how important (from ‘essential’ to ‘should not be included’) you think it is that each statement be included in the guidelines.

If the person does not want to change

* 56. The first aider should be aware that the Aboriginal person can only be assisted if they are ready to change their gambling behaviours. (Rerate)

- Essential
- Important
- Don't know/Depends
- Unimportant
- Should not be included

* 57. If the Aboriginal person does not want to change their gambling behaviours, the first aider should **sensitively** ask the Aboriginal person if gambling and its consequences are getting in the way of them living the life they want to live.(Rerate)

- Essential
- Important
- Don't know/Depends
- Unimportant
- Should not be included

* 58. The first aider should tell the Aboriginal person that they will be available to assist the Aboriginal person when they are ready to change their gambling behaviours. (Rerate)

- Essential
- Important
- Don't know/Depends
- Unimportant
- Should not be included

Reducing the negative impact

* 59. *<u>If the Aboriginal person decides to continue gambling</u>*, the first aider should encourage them to reduce the negative impact of gambling by:

	Essential	Important	Don't know/Depends	Unimportant	Should not be included
a. Limiting time spent on gambling. (Rerate)	◯	◯	◯	◯	◯
b. Limiting money spent on gambling. (Rerate)	◯	◯	◯	◯	◯
c. Only gambling with money that the Aboriginal person can afford to lose. (Rerate)	◯	◯	◯	◯	◯
d. Balancing time spent on gambling with other activities. (Rerate)	◯	◯	◯	◯	◯
e. Keeping a record of gambling wins and losses. (Rerate)	◯	◯	◯	◯	◯
f. Taking regular breaks while gambling. (Rerate)	◯	◯	◯	◯	◯
g. Leaving bank cards or credit cards at home. (Rerate)	◯	◯	◯	◯	◯

* 60. <u>If the Aboriginal person decides to continue gambling</u>, the first aider should discourage them from gambling with friends or family who have gambling problems. (Rerate)

◯ Essential

◯ Important

◯ Don't know/Depends

◯ Unimportant

◯ Should not be included

* 61. *If the Aboriginal person decides to continue gambling*, the first aider should encourage them to reduce the negative impact of gambling by:

	Essential	Important	Don't know/Depends	Unimportant	Should not be included
a. Not gambling to earn money or pay debts. (Rerate)	◯	◯	◯	◯	◯
b. Not trying to win back gambling losses. (Rerate)	◯	◯	◯	◯	◯
c. Not using borrowed money to gamble. (Rerate)	◯	◯	◯	◯	◯
d. Not using personal investments or savings to gamble. (Rerate)	◯	◯	◯	◯	◯

SECTION 6: Gambling first aid actions (cont.)

This section contains statements about what the first aider needs to do when providing assistance to an Aboriginal person with gambling problems.

Please keep our definitions in mind when completing the survey. You can access the definitions here.

Please rate how important (from 'essential' to 'should not be included') you think it is that each statement be included in the guidelines.

Assisting the person who wants to change

* 62. If the Aboriginal person decides to use self-help strategies, the first aider should offer to support them during this. (Rerate)

- ◯ Essential
- ◯ Important
- ◯ Don't know/Depends
- ◯ Unimportant
- ◯ Should not be included

* 63. The first aider should help the Aboriginal person make a list of strategies that can help them change their gambling behaviours. (Rerate)

- Essential
- Important
- Don't know/Depends
- Unimportant
- Should not be included

* 64. The first aider should **offer to assist** the Aboriginal person to list the strategies that can help them change their gambling behaviours. (New)

- Essential
- Important
- Don't know/Depends
- Unimportant
- Should not be included

* 65. The first aider should discuss with the Aboriginal person strategies that can help them change their gambling behaviours. (New)

- Essential
- Important
- Don't know/Depends
- Unimportant
- Should not be included

* 66. If the Aboriginal person is attempting to change their gambling behaviours, the first aider should focus on the future, rather than past mistakes. (Rerate)

- Essential
- Important
- Don't know/Depends
- Unimportant
- Should not be included

* 67. If the Aboriginal person is attempting to change their gambling behaviours, the first aider should note any positive behavioural changes and congratulate the Aboriginal person on these. (Rerate)

- Essential
- Important
- Don't know/Depends
- Unimportant
- Should not be included

* 68. If the Aboriginal person sets a budget and asks for help sticking to it, the first aider should support them in this. (Rerate)

- Essential
- Important
- Don't know/Depends
- Unimportant
- Should not be included

* 69. The first aider should encourage the Aboriginal person **to think about** the negative consequences of gambling, on a daily basis. (New)

- Essential
- Important
- Don't know/Depends
- Unimportant
- Should not be included

* 70. The first aider should encourage the Aboriginal person **to think about** the positive consequences of **not** gambling, on a daily basis. (New)

- Essential
- Important
- Don't know/Depends
- Unimportant
- Should not be included

* 71. The first aider should offer to assist the Aboriginal person to list the advantages and disadvantages of gambling. (New)

- Essential
- Important
- Don't know/Depends
- Unimportant
- Should not be included

* 72. The first aider should ask the Aboriginal person to tell a story about a positive day without gambling. (New)

- Essential
- Important
- Don't know/Depends
- Unimportant
- Should not be included

* 73. If the Aboriginal person is experiencing anxiety, anger, stress, depression or boredom, the first aider should provide social support, as these may be triggers for worsening of gambling problems. (Rerate)

- Essential
- Important
- Don't know/Depends
- Unimportant
- Should not be included

SECTION 6: Gambling first aid actions (cont.)

This section contains statements about what the first aider needs to do when providing assistance to an Aboriginal person with gambling problems.

Please keep our definitions in mind when completing the survey. You can access the definitions here.

Please rate how important (from ‘essential’ to ‘should not be included’) you think it is that each statement be included in the guidelines.

Assisting the person who wants to change (cont.)

* 74. *If asked by the Aboriginal person*, the first aider should discuss with them possible strategies for handling gambling urges and encourage the Aboriginal person to use them.

- Essential
- Important
- Don't know/Depends
- Unimportant
- Should not be included

* 75. The first aider should encourage the Aboriginal person to do the following:

	Essential	Important	Don't know/Depends	Unimportant	Should not be included
Reduce the amount of time and money spent on gambling. (Rerate)	○	○	○	○	○
Avoid spending time with people who are associated with gambling. (Rerate)	○	○	○	○	○
Share their successes in gambling lapses with supportive people. (Rerate)	○	○	○	○	○

* 76. If the Aboriginal person asks for the first aider's assistance to change their gambling, the first aider should offer:

	Essential	Important	Don't know/Depends	Unimportant	Should not be included
a. To approach an appropriate service to ask them to organise a gambling 'healing circle'. (New)	○	○	○	○	○
b. To approach an appropriate service to ask them to organise a gambling 'yarning circle'. (New)	○	○	○	○	○
c. To approach an appropriate service to organise a gambling forum. (New)	○	○	○	○	○
d. To have one-one conversations about their gambling with the first aider. (New)	○	○	○	○	○
e. To give them information about available services in the community. (New)	○	○	○	○	○

SECTION 6: Gambling first aid actions (cont.)

This section contains statements about what the first aider needs to do when providing assistance to an Aboriginal person with gambling problems.

Please keep our definitions in mind when completing the survey. You can access the definitions here.

Please rate how important (from ‘essential’ to ‘should not be included’) you think it is that each statement be included in the guidelines.

Financial strategies

* 77. In order to limit access to money for gambling, the first aider should ask the Aboriginal person to consider the following:

	Essential	Important	Don't know/Depends	Unimportant	Should not be included
a. Paying all critical household expenses before paying off gambling debts. (Rerate)	○	○	○	○	○
b. Voluntarily using a basics card which cannot be used for gambling or cash withdrawals. (Rerate)	○	○	○	○	○

* 78. If the Aboriginal person asks for money to help cover bills or debts, the first aider should refer them to a relief agency or financial counselling service, rather than giving them money. (Rerate)

- ○ Essential
- ○ Important
- ○ Don't know/Depends
- ○ Unimportant
- ○ Should not be included

* 79. If the Aboriginal person asks for money to help cover bills or debts, the first aider should consider giving them food instead, especially if children are affected. (New)

- ○ Essential
- ○ Important
- ○ Don't know/Depends
- ○ Unimportant
- ○ Should not be included

Section 6: Gambling first aid actions (cont.)

This section contains statements about what the first aider needs to do when providing assistance to an Aboriginal person with gambling problems.

Please keep our definitions in mind when completing the survey. You can access the definitions here.

Please rate how important (from ‘essential’ to ‘should not be included’) you think it is that each statement be included in the guidelines.

Concerns for safety

* 80. The first aider should be aware that suicidal thoughts and behaviours are more common in people with gambling problems. (Rerate)

- Essential
- Important
- Don't know/Depends
- Unimportant
- Should not be included

* 81. The first aider should be aware that the Aboriginal person may see suicide as a way to avoid difficult confrontations with loved ones or creditors. (Rerate)

- Essential
- Important
- Don't know/Depends
- Unimportant
- Should not be included

* 82. If the Aboriginal person is involved in illegal activities related to their gambling, the first aider should:

	Essential	Important	Don't know/Depends	Unimportant	Should not be included
a. Encourage the Aboriginal person to stop these activities. (Rerate)	○	○	○	○	○
b. Encourage the Aboriginal person to seek legal assistance. (Rerate)	○	○	○	○	○

Thank you.

That is the end of the Round 2 survey. Thank you for sharing your time and expertise with us.

If participating in this survey has caused you distress and you wish to talk to someone about this, you can contact Lifeline on 13 11 14.

By pressing the ‘Done’ button your responses will be registered with our survey software. Once all panel members have lodged their responses, we will collate the data and send you a report on the findings and the Round 3 survey.

Kind Regards,

The Mental Health First Aid Research Team

Assisting an Aboriginal or Torres Strait Islander person with gambling problems - R3

Introduction and Instructions

Purpose of this research
The purpose of this project is to develop a set of guidelines for the public on how to support an Aboriginal or Torres Strait Islander person with gambling problems. This project has received funding from the Australian Government Department of Health.

How this questionnaire was developed
The statements in this questionnaire were derived from the results of the Round 2 survey and consist of any of the new items that need to be rerated. An item is rerated when 80-89% of the panel members rated it as 'essential' or 'important'.

Instructions
Please complete the questionnaire by rating each statement according to how important you believe it is for inclusion in the guidelines for helping n Aboriginal person with gambling problems. Please keep in mind that the guidelines will be used by the **general public**. The statements need to be rated according to their importance for someone **without a counselling or clinical background**.

This questionnaire should take approximately 10 minutes to complete. You can complete the survey in two or more sittings. Your answers are saved when you click ‘Next’ at the bottom of a page. This marks your page and you can begin again at a later date on the next page. Please be aware that once you have logged on and started responding you must complete the questionnaire on the same computer.

IMPORTANT NOTE:

There are already guidelines on cultural considerations and communication techniques (click here to access these guidelines) for providing mental health first aid to an Aboriginal person who may be experiencing a range of mental health problems (access the full range of guidelines). **We do not wish to replicate these guidelines**. Rather, our aim is to develop guidelines on how to assist an Aboriginal person who is experiencing gambling problems.

Structure of this survey
This survey is divided into the following section:
SECTION 1: Cultural considerations when assisting an Aboriginal person with gambling problems
SECTION 2: Warning signs of a gambling problem
SECTION 3: Awareness about gambling and gambling problems
SECTION 4: Awareness about gambling treatment and recovery from gambling problems
SECTION 5: Preparing to talk with the person
SECTION 6: Assisting the person who wants to change

Aboriginal in this questionnaire refers to Aboriginal or Torres Strait Islander people.

First aider refers to a concerned family member, friend, work colleague, or work supervisor who provides assistance to an Aboriginal person with gambling problems.

The Aboriginal person refers to the Aboriginal or Torres Strait Islander person with gambling problems or suspected gambling problems.

Gambling is the staking of money on uncertain events that are driven by chance.

Gambling problems are difficulties over time in limiting money or time spent on gambling, which leads to adverse consequences for the person, others, or for the community. This could include someone whose gambling problems are at a clinically diagnosable level.

Venue refers to a virtual or land-based location offering gambling or gaming activities with the chance to win money.

Gambling first aid is the assistance given to the Aboriginal person who is developing a gambling problem, experiencing a worsening of their gambling problem or experiencing a mental health crisis related to gambling. The assistance is given until appropriate professional help is received or until the crisis resolves.

Assisting an Aboriginal or Torres Strait Islander person with gambling problems - R3

There are already guidelines on cultural considerations and communication techniques (click here to access these guidelines) for providing mental health first aid to an Aboriginal person who may be experiencing a range of mental health problems (access the full range of guidelines). We do not wish to replicate these guidelines. Rather, our aim is to develop guidelines on how to assist an Aboriginal person who is experiencing gambling problems.

Please keep our definitions in mind when completing the survey. You can access the definitions here.

Please rate how important (from 'essential' to 'should not be included') you think it is that each statement be included in the guidelines.

* 1. Please provide us with your email address. (This allows us to track who completes the Round 2 survey and is then eligible to complete Round 3.)

Cultural considerations when assisting an Aboriginal person with gambling problems

* 2. The first aider should know how the expectation of 'providing for family' may impact on gambling problems, e.g. other family members may provide for the person if they lose their money for rent or food.

- ◯ Essential
- ◯ Important
- ◯ Don't know/Depends
- ◯ Unimportant
- ◯ Should not be included

Warning signs

* 3. The first aider should be aware that the following behavioural signs indicate that an Aboriginal person may have gambling problems:

	Essential	Important	Don't know/Depends	Unimportant	Should not be included
The person moves from community gambling (e.g. cards) to commercial gambling (e.g. pokies). (New)	◯	◯	◯	◯	◯

Awareness about gambling problems

* 4. The first aider should try to learn more about gambling problems, e.g. ring a gambling help line, talking with others, reading information.

◯ Essential

◯ Important

◯ Don't know/Depends

◯ Unimportant

◯ Should not be included

Awareness about treatment resources

* 5. The first aider should **offer** to make an appointment for the person with a gambling help or other service.

◯ Essential

◯ Important

◯ Don't know/Depends

◯ Unimportant

◯ Should not be included

Preparing to talk with the person

* 6. The first aider should know that it may be a long-term investment to support an Aboriginal person with gambling problems, particularly if the first aider is not Aboriginal.

- ◯ Essential
- ◯ Important
- ◯ Don't know/Depends
- ◯ Unimportant
- ◯ Should not be included

Assisting the person who wants to change

* 7. The first aider should discuss with the Aboriginal person strategies that can help them change their gambling behaviours.

- ◯ Essential
- ◯ Important
- ◯ Don't know/Depends
- ◯ Unimportant
- ◯ Should not be included

* 8. The first aider should encourage the Aboriginal person **to think about** the positive consequences of **not** gambling, on a daily basis.

- ◯ Essential
- ◯ Important
- ◯ Don't know/Depends
- ◯ Unimportant
- ◯ Should not be included

* 9. The first aider should offer to assist the Aboriginal person to list the advantages and disadvantages of gambling.

- ◯ Essential
- ◯ Important
- ◯ Don't know/Depends
- ◯ Unimportant
- ◯ Should not be included

That is the end of the Round 3 survey. Thank you for sharing your time and expertise with us.

If participating in this survey has caused you distress and you wish to talk to someone about this, you can contact Lifeline on 13 11 14.

By pressing the ‘Done’ button your responses will be registered with our survey software. Once all panel members have lodged their responses, we will use the results to write the guidelines. We will send a final draft of the guidelines for your final endorsement.

Kind Regards,

The Mental Health First Aid Research Team
